# Legacy Metal Contaminants and Excess Nutrients in Low Flow Estuarine Embayments Alter Composition and Function of Benthic Bacterial Communities

**DOI:** 10.3389/fmicb.2021.661177

**Published:** 2021-10-08

**Authors:** Simone C. Birrer, Franziska Wemheuer, Katherine A. Dafforn, Paul E. Gribben, Peter D. Steinberg, Stuart L. Simpson, Jaimie Potts, Peter Scanes, Martina A. Doblin, Emma L. Johnston

**Affiliations:** ^1^Evolution and Ecology Research Centre, School of Biological, Earth and Environmental Sciences, University of New South Wales, Kensington, NSW, Australia; ^2^Sydney Institute of Marine Science, Mosman, NSW, Australia; ^3^Department of Earth and Environmental Sciences, Macquarie University, North Ryde, NSW, Australia; ^4^Centre for Marine Science and Innovation, School of Biological, Earth and Environmental Sciences, University of New South Wales, Kensington, NSW, Australia; ^5^CSIRO Land and Water, Centre for Environmental Contaminants Research, Canberra, ACT, Australia; ^6^Coastal Waters Unit, Science Division, NSW Department of Planning, Industry and Environment, Sydney, NSW, Australia; ^7^Climate Change Cluster, University of Technology, Sydney, NSW, Australia

**Keywords:** hydrology, sediment, bacteria, 16S rRNA, RNAseq, multiple stressors, estuary

## Abstract

Coastal systems such as estuaries are threatened by multiple anthropogenic stressors worldwide. However, how these stressors and estuarine hydrology shape benthic bacterial communities and their functions remains poorly known. Here, we surveyed sediment bacterial communities in poorly flushed embayments and well flushed channels in Sydney Harbour, Australia, using 16S rRNA gene sequencing. Sediment samples were collected monthly during the Austral summer-autumn 2014 at increasing distance from a large storm drain in each channel and embayment. Bacterial communities differed significantly between sites that varied in proximity to storm drains, with a gradient of change apparent for sites within embayments. We explored this pattern for embayment sites with analysis of RNA-Seq gene expression patterns and found higher expression of multiple genes involved in bacterial stress response far from storm drains, suggesting that bacterial communities close to storm drains may be more tolerant of localised anthropogenic stressors. Several bacterial groups also differed close to and far from storm drains, suggesting their potential utility as bioindicators to monitor contaminants in estuarine sediments. Overall, our study provides useful insights into changes in the composition and functioning of benthic bacterial communities as a result of multiple anthropogenic stressors in differing hydrological conditions.

## Introduction

Estuaries are diverse and productive ecosystems that support many human activities. However, the diversity and functioning of these ecosystems are increasingly threatened by multiple anthropogenic stressors including contamination and habitat modification (Johnston et al., 2015a; Mayer-Pinto et al., 2017; Vadillo Gonzalez et al., 2019). Industrial contamination and pulses of stormwater carrying urban contaminants are some of the greatest threats to estuarine health and stability. Upon entering the waterway, the distribution and concentrations of contaminants in estuaries is largely dependent on hydrological conditions (Birch and Rochford, 2010; Mayer-Pinto et al., 2015; Machado et al., 2016; Nystrand et al., 2016). Specifically, under low flow conditions, any contaminants entering a waterway are likely to be retained for longer compared to the rapid transit and dilution that occur under high flow conditions (Sutherland et al., 2017). Hotspots of contamination have been documented where low flow estuarine environments exist naturally, such as at the ends of embayments (Birch and Rochford, 2010; Sutherland et al., 2017), or those created by built structures such as break-walls surrounding marinas (Floerl and Inglis, 2003; Johnston, 2011; Sim et al., 2015). Understanding the consequences of these hydrological differences for the distribution and potential impacts of contaminants is important to manage the health and stability of estuarine ecosystems.

Bacterial communities play a key role in ecosystem stability and functioning (Falkowski et al., 2008). Moreover, several bacteria are important for the biodegradation of contaminants such as excess nutrients, or petroleum hydrocarbons and speciation of metals (Hammack and Edenborn, 1992; Das and Chandran, 2011). Allison and Martiny (2008) proposed that changes in microbial communities in response to different environmental disturbances may directly influence ecosystem functions. Due to their importance for ecosystem function and their sensitivity to altered environmental conditions, bacterial communities can be used to monitor environmental perturbations (Sun et al., 2012; Aylagas et al., 2017; Glasl et al., 2017). It is therefore important to identify factors that influence bacterial communities and, as a result, alter the ecosystem functions they provide (Sun et al., 2013).

In the past decade, bacterial communities in coastal sediments and their responses toward anthropogenic stressors have received significant attention (Liu et al., 2014; Lawes et al., 2017; Beale et al., 2018; Birrer et al., 2018; Su et al., 2018). For instance, significant changes in bacterial community composition and predicted functions in surface sediments of Hangzhou Bay (China) in response to nutrients and metal contaminants were recorded by Su et al. (2018). In another study, the community composition and richness of benthic bacterial communities in chemically polluted sites along the coast of Italy were altered by polychlorinated biphenyls (PCBs), polycyclic aromatic hydrocarbons (PAHs) and metals (Quero et al., 2015). Sun et al. (2013) investigated the response of sediment bacterial communities to contaminant disturbance across six estuaries with differing levels of modification along the coast of New South Wales, Australia. They observed that sediment metals and silt content explained most of the variation seen in the community composition, while PAHs explained little of the variance. However, functional responses of bacterial communities in estuarine sediments to multiple urban stressors (e.g., metals and excess nutrients) are still not well-understood, as many previous studies have focused on community changes only (but see Lu et al., 2017; Birrer et al., 2018; Su et al., 2018).

The aim of the present study was to investigate how the diversity, community composition and functions of benthic bacteria in Sydney Harbour (Australia) change in relation to multiple contaminants and estuarine hydrology. Sydney Harbour is one of the largest estuaries in the world and a hotspot for biological diversity (Johnston et al., 2015b; Mayer-Pinto et al., 2015). In a previous study, Sutherland et al. (2017) assessed contaminant hotspots and biogeochemical processes in sediment communities at four locations in Sydney Harbour that were either poorly flushed (low flow and high retention) embayments or well-flushed (high flow and low retention) channels. For this purpose, sediment samples were taken monthly from two embayment and two channel locations at increasing distances (approximately 0, 200, and 1,000 m away) from a large storm drain. The authors observed significant differences in biogeochemical processes between the two retention types and changes in benthic metabolism with distance from storm drains. They suggested that embayments are particularly vulnerable to the negative effects of contaminant retention, while the apparent resilience of fast-flowing channels may mean that these locations are more suitable for handling stormwater inputs due to the potential for rapid dilution and transit.

In the present study, we assessed benthic bacterial communities by Illumina (MiSeq) sequencing targeting the bacterial 16S rRNA genes using the same sediment samples analysed by Sutherland et al. (2017). We further examined differences in gene expression patterns and in the composition of the potentially active bacterial community targeting embayment sediments at sites close to and far from storm drains by RNA-Seq analysis. Our main hypotheses were that bacterial alpha diversity measures and community composition differ between channels and embayments, and bacterial community structure and function in embayment systems differ depending on proximity to storm drains.

## Materials and Methods

### Sampling Design

**Figure 1:**
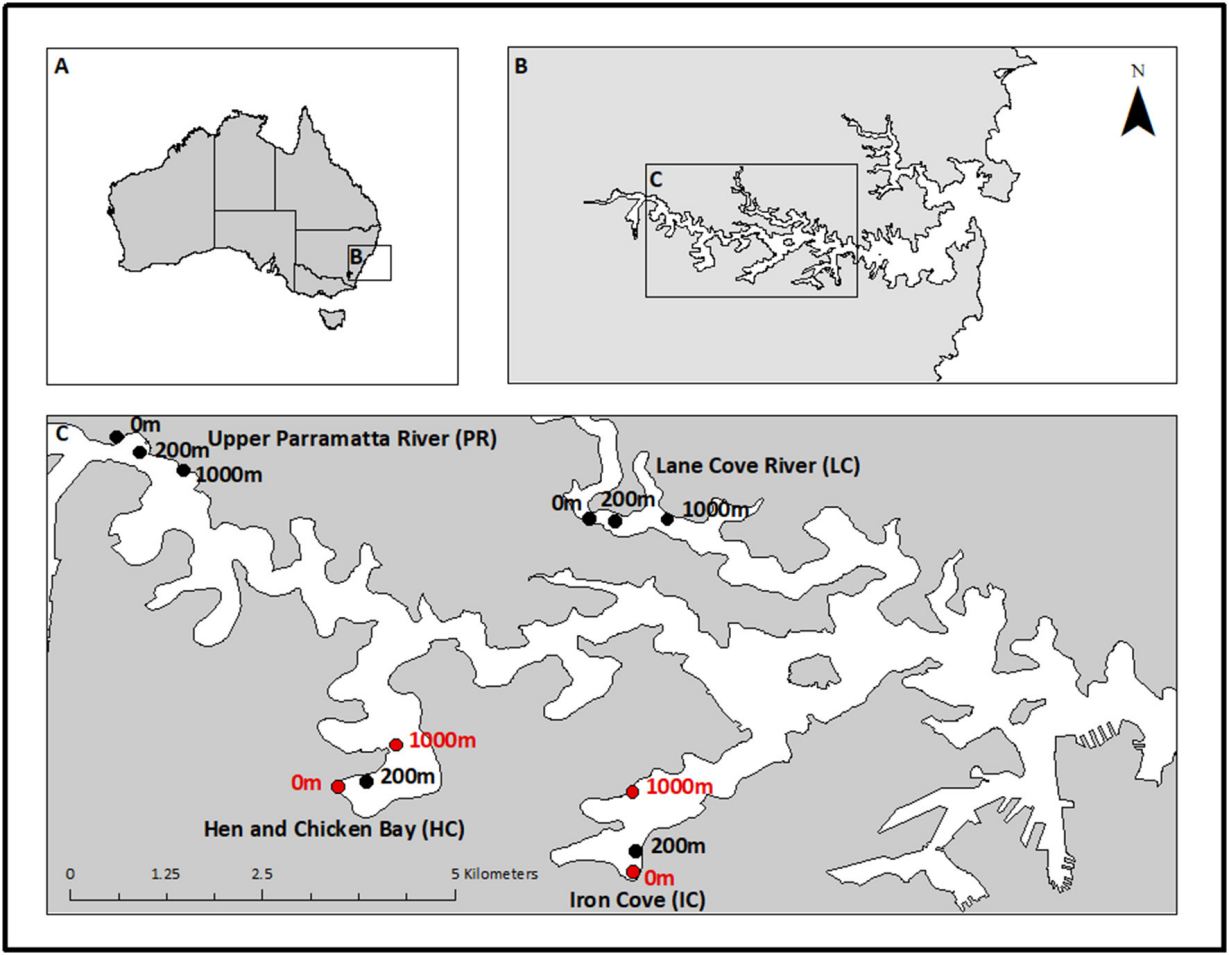
Sampling locations in Sydney Harbour, New South Wales, Australia. Map shows **(A)** Australia, **(B)** Sydney Harbour estuary, and **(C)** locations sampled within the estuary. The Upper Parramatta River (100 km^2^; PR) and Lane Cove River (80 km^2^; LC) locations are well flushed channels with low retention. The embayments Hen and Chicken Bay (< 10 km^2^; HC) and Iron Cove (14 km^2^; IC) experience very little flushing and were therefore selected as high retention sites. Samples were taken monthly from February to May 2014. Sampling sites within each location (*n* = 3) are indicated by approximate proximity to a large storm drain (i.e., 0, 200, 1,000 m). Sites highlighted in red were selected for metatranscriptomics analysis (collected in February and March).

Sediment samples were collected monthly in the Austral summer-autumn between February 2014 and June 2014 (four sampling times) in Sydney Harbour (Port Jackson; 33°50‘S, 151°15‘E, Figure 1). Sydney Harbour is a drowned river valley (Roy et al., 2001) situated on the temperate south-east coast of New South Wales, Australia. It comprises a complex network of embayments and inlets connected through channels adjacent to a winding and increasingly larger channel opening to the Tasman Sea. Over the past 200 years, human activities including the dredging of shipping channels and land reclamation have extensively modified the bathymetry and shoreline of Sydney Harbour (Birch et al., 2009). Past urban stormwater inflows and historical industrial practices have left a legacy of contamination in surrounding sediments (Chariton et al., 2010; Dafforn et al., 2012).

Four locations in the central portion of Sydney Harbour were randomly selected to represent different hydrology and water retention (Figure 1). Two locations were in well-flushed channels with low retention (Lane Cover River and Upper Parramatta River) and two locations were in poorly flushed embayments with high retention (Hen and Chicken Bay and Iron Cove River). The embayments contain high concentrations of contaminants (Irvine and Birch, 1998). Within each location, sampling was done at increasing distances from major stormwater outlets (0, 200, and 1,000 m), representing a potential gradient away from a contaminant hotspot. The three distances were selected based on hydrodynamic modelling by Lee et al. (2011) who described freshwater-plume penetration into the Sydney Harbour estuary during significant rainfall events (> 50 mm/day).

### Sample Collection and Chemical Analyses

Sediment samples were collected during relatively dry conditions (<5 mm rainfall/day, Bureau of Meteorology, 2014). As a consequence, ongoing stormwater inputs into the estuary were minimal (<0.1 m^3^/s) (Birch and Rochford, 2010). The sampling and chemical analyses were described in detail by Sutherland et al. (2017). In brief, surficial sediments (<5 cm depth) were collected subtidally using a Van Veen sediment grab from a water depth of approximately 1.5–2.5 m at each distance. Two sediment grabs per distance were collected approximately 3 m apart and sub-sampled for the surface (<2 mm depth) microbial community in sterile, DNase- and RNase-free 2 mL cryo tubes. The surface 2 mm of sediment was sampled for the bacteria to target the most oxygenated layer that would be in contact with both the water and sediment to allow for comparisons with physicochemical measures collected from both media. To ensure that the transcription was directly related to environmental conditions, sediment samples for molecular analyses were immediately frozen in liquid nitrogen in the field. Samples remained in liquid nitrogen until returning to the laboratory and were then stored at −80°C until extractions were performed within 4 weeks of sampling.

The remaining grab sample was then homogenised in a clean tray and sub-sampled for metal, carbon, nitrogen and grain size analyses. Sediments from the whole grab (i.e., 0–5 cm depth) were used to determine sediment properties because a greater quantity is required compared to the microbial analysis. Plasticware used to collect sediment for metal analyses was previously soaked in 5% HNO_3_ for a minimum of 24 h and then rinsed in deionised water (Milli-Q™). Samples were kept in the dark on ice for transport to the laboratory. Samples for chemical analyses were frozen at −20°C. In brief, total organic carbon and total nitrogen were analysed at Isoenvironmental (South Africa) using 5–20 mg of dried, homogenised sample in a 20-20 IRMS linked to an ANCA SL element analyser (Europa Scientific). Metal analyses followed Dafforn et al. (2012). Sediments were oven-dried (50°C) and homogenised to a fine powder with mortar and pestle before microwave digestion according to method 3051A (USEPA, 2007). Metal concentrations were analysed using ICP-AES (Perkin Elmer, OptimaOptima7300DV, USA). Grain size analyses were performed on wet sediments using a Malvern laser particle size analyser (Mastersizer 2000, USA) and the percentage of silt content (% <63 μm) was calculated. All environmental properties are provided as Supplementary Table 1.

### Extraction and Purification of Nucleic Acids

Environmental RNA and DNA were extracted from 2 g of wet sediment using the RNA PowerSoil® Total RNA Isolation Kit and the RNA PowerSoil® DNA Elution Accessory Kit (Mo Bio Laboratories Inc., Carlsbad, CA, USA, now Qiagen), respectively, according to the manufacturer recommendations (http://www.qiagen.com, accessed 25/01/21). Extracted DNA was purified using Agencourt AMPure XP (Agencourt Bioscience Corporation, Beverly, MA, USA) following manufacturer recommendations (https://www.beckmancoulter.com/wsrportal/techdocs?docname=B37419, accessed 25/01/21). DNA concentrations of all purified samples were quantified using a NanoDrop ND-1000 spectrophotometer (NanoDrop Technologies, Wilmington, DE, USA). To remove residual DNA from RNA samples, the TURBO DNA-free^TM^ Kit (Life Technologies, Carlsbad, CA, USA) was used following manufacturer recommendations for routine DNase treatment (https://assets.thermofisher.com/TFS-Assets/LSG/manuals/1907M_turbodnafree_UG.pdf, accessed 21/01/21). Subsequently, RNA was purified with the Agencourt® RNAClean® XP (Agencourt Bioscience Corporation, Beverly, MA, USA). The RNA integrity number (RIN) was measured for each sample to control for RNA degradation. The mean RIN for all samples was 7.72, where a RIN of 10 represents no degradation.

The absence of DNA in RNA samples was confirmed by PCR using the primers 515FB (5′-GTGYCAGCMGCCGCGGTAA-3′) and 806RB (5′-GGACTACNVGGGTWTCTAAT-3′) from the earth microbiome project (https://earthmicrobiome.org/protocols-and-standards/16s/, accessed 25/01/21). The PCR reaction mixture (50 μL) contained 10 μL of five-fold MyTaq Reaction Buffer (Bioline, Alexandria, NSW), 0.4 μM of each primer, 1 U of MyTaq DNA Polymerase (Bioline, Alexandria, NSW), and approximately 10 ng of purified RNA as template. The following thermal cycling scheme was used: initial denaturation at 95°C for 2 min, 28 cycles of denaturation at 95°C for 30 s, annealing at 50°C for 30 s, followed by extension at 72°C for 1 min. The final extension was carried out at 72°C for 2 min. Negative controls were performed by using the reaction mixture without template. Genomic DNA from *Escherichia coli* DH5α was used in the positive control. The success of the PCR was controlled by gel electrophoresis. No PCR signals were detected for the RNA samples.

### Sequencing and Processing of the 16S rRNA Gene Data

Bacterial communities in sediments were assessed by paired-end sequencing targeting the 16S rRNA gene at the Molecular Research DNA (MRDNA) Lab (https://www.mrdnalab.com, Shallowater, TX, USA). The V2-V3 region was amplified with primers 104F (Bertilsson et al., 2002) and 530R (Lane, 1991) for consistency with our previous research to provide comparable results to the bacterial diversity investigations undertaken in the same estuary (Sun et al., 2013). PCRs were done using the HotStarTaq *Plus* Master Mix Kit (Qiagen, Valencia, CA, USA) under the following conditions: 94°C for 3 min, followed by 28 cycles of 94°C for 30 s, 53°C for 40 s, and 72°C for 1 min, after which a final elongation step at 72°C for 5 min was performed. After amplification, PCR products were checked in 2% agarose gel to determine the success of amplification and the relative intensity of bands. Samples were pooled together in equal proportions based on their molecular weight and DNA concentrations. Pooled samples were purified using calibrated Agencourt AMPure XP beads (Agencourt Bioscience Corporation, Beverly, MA, USA). The purified PCR products were used to prepare DNA library by following Illumina TruSeq DNA library preparation protocol using the MiSeq reagent kit V2 (Illumina Inc., San Diego, CA, USA). Sequencing was performed at the MRDNA lab (https://www.mrdnalab.com, Shallowater, TX, USA) on a MiSeq Sequencing platform (Illumina Inc., San Diego, CA, USA) following the manufacturer recommendations.

Obtained sequencing data were initially quality filtered with the Trimmomatic tool version 0.36 (Bolger et al., 2014). Low quality reads were truncated if the quality dropped below 10 in a sliding window of 4 bp. Subsequently, all reads shorter than 100 bp and orphan reads were removed. Remaining sequences were subsequently processed with USEARCH version 10.240 (Edgar, 2010). Paired-end reads were merged and quality-filtered. Filtering included the removal of reads shorter than 350 bp or longer than 470 bp as well as the removal of low-quality reads (maximum number of expected errors >1 or 1 ambiguous base). Processed sequences of all samples were concatenated into one file and subsequently dereplicated into unique sequences. Obtained unique sequences were denoised and clustered into zero-radius operational taxonomic units (zOTUs) with the *unoise3* algorithm implemented in USEARCH (Edgar, 2010).

Chimeric sequences were removed *de novo* using the UCHIME algorithm during clustering (Edgar et al., 2011). Additionally, the *unoise3* algorithm removed all unique sequences which appeared <8 times in the entire data set. Remaining chimeric sequences were removed using UCHIME (Edgar et al., 2011) in reference mode with the SILVA SSU Ref NR 99 132 database (Quast et al., 2013). To assign taxonomy of bacteria, unique and chimaera-free sequences were classified by BLAST alignment (Camacho et al., 2009) against the SILVA database with an e value cutoff of 1e-20. Processed sequences were mapped onto zOTU sequences to calculate the distribution and abundance of each zOTU in every sample using the *otutab* command with *maxrejects* and *maxaccepts* options disabled. All non-bacterial zOTUs were removed based on their taxonomic classification in the SILVA database. In addition, all zOTUs consisting of one single sequence (singletons) were removed prior to statistical analysis. The final OTU table is provided as Supplementary Table 2. Sequence characteristics are provided for each sample in Supplementary Table 3.

### Sequencing and Processing of Metatranscriptomic Data

Based on patterns in the bacterial community composition observed from the amplicon sequencing, sediment samples collected at 0 and 1,000 m from the storm drain in the embayments at two sampling times (Feb/Mar 2014, *n* = 16) were selected for RNA sequencing. RNA libraries with fragment lengths of approximately 200 bp were prepared. Prior to library preparation, the quality of the total RNA samples was assessed on a Bioanalyzer 2100 using an RNA 6000 Nano Chip (Agilent Technologies, Santa Clara, CA, USA). Sample quantitation was carried out using Invitrogen's Ribogreen assay (Invitrogen, Carlsbad, Ca, USA). Library preparation was then performed according to the TruSeq Stranded mRNA protocol (Illumina Inc., San Diego, CA, USA) with the following modifications: the oligo-dT mRNA purification step was omitted and instead, 200 ng of total RNA were directly added to the Elution2-Frag-Prime step. The amplification step of the PCR was performed according to the manufacturer's recommendations, but the number of amplification cycles was reduced to 12. Each library was uniquely tagged with one of Illumina's TruSeq LT RNA barcodes and quantified using Invitrogen's Picogreen assay (Invitrogen, Carlsbad, Ca, USA). The average library size was determined on a Bioanalyzer 2100, using a DNA 7500 chip (Agilent Technologies, Santa Clara, CA, USA). Library concentrations were then diluted to 2 nM, and the concentrations were validated by qPCR on a ViiA-7 real-time thermocycler (Applied Biosystems, Foster City, CA, USA), using qPCR primers recommended in Illumina's qPCR protocol, and Illumina's PhiX control library as standard. The libraries were then pooled at equimolar concentrations and sequenced across two lanes on an Illumina HiSeq2500 sequencer in rapid mode at a read-length of 100 bp paired-end as recommend by the manufacturer. Sequencing was performed at the Singapore Centre for Environmental Life Sciences Engineering (SCELSE).

Generated sequencing data were initially quality filtered and residual adaptor sequences were removed with Trimmomatic version 0.36 (Bolger et al., 2014). The quality of the RNA data and the absence of adaptor sequences were controlled using FastQC version 0.11.7 (Andrews, 2010). Ribosomal RNA (rRNA) was removed *in silico* using SortMeRNA version 2.1 (Kopylova et al., 2012). Residual sequences were assembled using rnaSPAdes version 3.12.0 (Bankevich et al., 2012). The occurrences and abundance of each assembled transcript was determined by mapping the unassembled reads on the assembled transcripts using Bowtie version 2.3.2 (Langmead and Salzberg, 2012) with RSEM version 1.3.0 (Ferrari et al., 2011). Because RSEM cannot handle paired and unpaired data simultaneously, they were mapped individually, and the mean expected count was calculated from both assemblies taking the numbers of paired and unpaired reads into account. mRNA reads of all samples were assembled together and individual reads of each sample were mapped on obtained contigs. As we only compared transcription levels of individual genes between samples, gene length did not differ and thus was not considered in the analysis.

Prodigal version 2.6.3 (Hyatt et al., 2010) was used to predict open reading frames (ORFs) and protein sequences. Predicted protein sequences were functionally annotated using UPRoC version 1.2 (Meinicke, 2014) and the KEGG database (Kanehisa, 2002) implemented in UPRoC. To taxonomically classify assembled transcripts, we used Kaiju version 1.6.2 (Menzel et al., 2016) and the NCBI NR release with eukaryotic sequences (2018-02-23). Only bacterial transcripts were considered in the functional analysis. To obtain a deeper, PCR-unbiased insight into the active community, rRNA sequences identified with SortMeRNA were mapped on unique and chimaera-free sequences obtained from the 16S rRNA amplicon data using bowtie2 (Langmead and Salzberg, 2012). The final zOTU and transcript tables for the active bacterial community dataset can be found in Supplementary Table 4.

### Statistical Analyses

All statistical analyses were performed in R version 3.4.0 (R Core Team, 2018) as well as in PRIMER 7 with the PERMANOVA add-on (PRIMER-E, Plymouth Marine Laboratory, UK). Differences were considered as statistically significant with *p* ≤ 0.05.

Environmental data (water and sediment characteristics) were normalised prior to statistical analyses. Multivariate datasets were initially explored with pairwise correlations between variables (Draftsman Plot). Where variables were strongly correlated (*r* > 0.9), a single variable was selected as representative for further analyses (e.g., Zn was strongly correlated with Al, As, Cd, Fe, Ni, Pb). Principal Components Analysis (PCA) was performed on normalised data to visualise relationships between retention type and proximity to storm drain as well as their interaction on environmental properties.

Differences in sequencing depth were tested by Kruskal-Wallis test. There were no significant differences in library size among distances, locations or sampling month. Hence, data were not normalised prior to further analyses (except for the alpha diversity analysis). Alpha diversity indices (richness, Shannon index of diversity, Faith's phylogenetic diversity, Chao1 and Michaelis-Menten Fit) were calculated using the R packages *vegan* 2.4.-4 (Oksanen et al., 2017), *picante* version 1.7 (Kembel et al., 2010) and *drc* version 3.0-1 (Ritz et al., 2015). Sample coverage was estimated using the Michaelis-Menten Fit calculated in R (Hughes et al., 2001). For this purpose, richness and rarefaction curves were calculated using the *specnumber* and *rarecurve* function, respectively, in *picante*. The Michaelis-Menten Fit was subsequently calculated from generated rarefaction curves using the *MM2* model within the *drc* package. Faith's phylogenetic diversity was generated using Fasttree version 2.1.10 (Price et al., 2010). Prior to tree calculation, sequences were aligned using PyNAST against the aligned version of the SILVA database. All alpha diversity indices were calculated 10 times. The OTU tables were rarefied to 29,919 sequences in each iteration using the *rrarefy* function in *vegan*. The average of all iterations was used for further statistical analyses. Bacterial alpha diversity values are provided as Supplementary Table 3.

Changes in alpha diversity values, environmental properties and abundant bacterial families/genera were evaluated by linear mixed effects models using the R packages *lmerTest* version 3.0-1 (Kuznetsova et al., 2018) and *MuMIn* version 1.42.1 (Barton, 2018). As fixed effects, we entered retention type (two levels: channel, embayment) and proximity to storm drain (three levels: 0, 200, 1,000 m) as well as their interaction term into each model. The random factors considered in each model were sampling month (four levels: February, March, April, May) and location (nested within retention: LC, PR, HC, IC). The exception was for water properties, where months were the replicates. A reduced model including proximity to storm drain (0 and 1,000 m) and location (HC and IC) was applied to abundant bacterial genera from the entire (amplicon) and active (rRNA) datasets. The best model was chosen according to lowest Akaike information criterion (AIC). The sample size was relatively small in comparison to the number of estimated parameters. As a consequence, AICc was used for model selection in the R package *MuMIn* (2018). Visual inspection of residual plots did not reveal any obvious deviations from homoscedasticity or normality. The final model was calculated using the function *lmer* provided within “lmerTest” with restricted maximum likelihood.

*P*-values were obtained by likelihood ratio tests of the full model against models without each of the fixed effects. Significance levels for fixed factors and their interaction are based on *F*-values, calculated by a type III analysis of variance with Satterthwaite approximation for degrees of freedom within the R package *lmerTest* (Kuznetsova et al., 2018). Statistically significant results were followed up with Tukey's HSD comparison tests, with *p*-value adjustment (*p* < 0.01) due to multiple comparisons. For random factors, an ANOVA-like table with likelihood ratio test statistics was generated using the *ranova* function in R package *lmerTest* (Kuznetsova et al., 2018).

Overall patterns of the entire bacterial community composition were analysed by permutational multivariate analysis of variance (PERMANOVA; Typ III) with 999 random permutations using the PRIMER 7 statistical package with the PERMANOVA + add-on (PRIMER-E, Plymouth Marine Laboratory, UK). The factors considered in each model were retention type (two levels: channel, embayment), proximity to storm drain (three levels: 0, 200, 1,000 m) as well as their interaction term, and sampling month (two levels: February, March) and location (nested within retention: HC, IC). analyses were done with Bray-Curtis dissimilarity measures. Differences in community composition among the distance gradient in each retention type (retention type ^*^ proximity to storm drain) were tested using pairwise tests. Bacterial zOTUs were analysed with the environmental properties using distance-based linear modelling (DistLM). *R*^2^ selection criteria and all specified selection procedure were used in the analysis. Results were visualised with distance-based redundancy analysis (dbRDA).

Potential differences in the composition of the active bacterial community as well as of transcripts were investigated by PERMANOVA with 1000 random permutations using the *vegdist* and *adonis* function within the *vegan* package in R using Bray-Curtis dissimilarities. Differences in community composition were tested using pairwise PERMANOVA (https://github.com/bwemheu/pairwise.adonis). Transcripts being differently expressed between the two investigated distances were detected using DESeq2 (Love et al., 2014). Prior to testing for differential abundance, an independent philtre was used to exclude genes absent with <10 counts in the entire data set. We controlled the false discovery rate using the *Benjamini–Hochberg* procedure for multiple comparisons (FDR = 0.1).

To identify bacterial zOTUs that were differentially associated with sediments close to or far from storm drains in embayments, multi-pattern analyses were applied with functions *multipatt* and *r.g* from the *indicspecies* package (De Cáceres and Legendre, 2009). To enhance reliability of the indicator analysis, only bacterial zOTUs found in at least two samples and with an abundance ≥ 0.001 in the active bacterial community were considered. In addition, we performed this analysis for the entire bacterial community using the same samples as for the metatranscriptomic analysis. The results of the indicator analysis with regard to the distance gradient are provided as Supplementary Table 5.

### Sequence Data Deposition

Sequence data were deposited in the sequence read archive (Ghosh et al., 2006) of the National Center for Biotechnology Information (NCBI) under accession numbers SUB7398817 (amplicons) and SUB7403872 (metatranscriptomes).

## Results

### General Sediment Characteristics

To investigate the effect of multiple anthropogenic stressors and estuarine hydrology on benthic bacterial communities, a total of 96 sediment samples were collected in embayments and channels once a month in the Austral summer-autumn between February 2014 and June 2014 (Figure 1 and Supplementary Table 1). Environmental properties were checked for co-linearity with a Draftsman Plot and the dataset was reduced to include temperature, salinity, Co, Cr, Cu, Ni, Zn (as representative of Al, As, Cd, Fe, Pb), %TOC, %TN and silt content. We analysed the effect of retention type and proximity to storm drain as well as their interaction on water and sediment properties with linear mixed-effect models (Table 1) and visualised these differences with Principal Components Analysis (PCA) ordination (Supplementary Figure 1 and Supplementary Table 1). The first two axes of the PCA ordination explained 63.4% of the total variance. PC1 explained 45.6%, mainly in relation to higher metal concentrations and silt content in the sediments 0 m and 200 m away from storm drains in embayments. PC2 explained 17.8%, mainly in relation to higher %TOC and %TN in embayments compared to channels. Channel sediments and those from embayments collected at 1,000 m from storm drains clustered together.

**Table 1:** Environmental characteristics (mean ± sd).

	**Embayment**			**Channel**		
	**0 m**	**200 m**	**1,000 m**	**0 m**	**200 m**	**1,000 m**
Co (mg/kg)	6.5 ± 3.2^a^	6.8 ± 2.2^a^	3.4 ± 1.6^b^	5.6 ± 2.8^ab^	3.8 ± 3.2^b^	5.2 ± 2.2^ab^
Cr (mg/kg)	61.5 ± 31.8^ab^	99.1 ± 55.9^a^	46 ± 40.2^b^	53.9 ± 55.5^ab^	46.2 ± 54.2^b^	55.1 ± 45.5^ab^
Cu (mg/kg)	238 ± 119^a^	323 ± 166^a^	124 ± 122^b^	60.6 ± 34.3^b^	42.4 ± 32.5^b^	73.5 ± 29.2^b^
Ni (mg/kg)	16.5 ± 8.2^a^	16.2 ± 6.8^a^	6.4 ± 4^b^	10.3 ± 5.4^ab^	7.1 ± 6.1^b^	9.8 ± 4.9^b^
Zn (mg/kg)	920 ± 580^a^	819 ± 406^a^	251 ± 142.8^b^	288 ± 220^b^	197 ± 173^b^	293 ± 157^b^
TN (%)	0.3 ± 0.1^a^	0.3 ± 0.1^a^	0.1 ± 0.1^b^	0.1 ± 0.1^b^	0.1 ± 0.1^b^	0.2 ± 0.1^b^
TOC (%)	6.4 ± 2.3^a^	4.3 ± 1.3^ab^	1.7 ± 0.7^c^	2.8 ± 1.3^bc^	2.6 ± 1.9^bc^	4 ± 4^b^
Silt content [<63 μm (%)]	72.2 ± 22.7^c^	97 ± 2.2^a^	79.8 ± 14.5^bc^	78.8 ± 15.1^bc^	80.7 ± 15.7^bc^	89.4 ± 8.6^ab^
Temperature (°C)	22.2 ± 3.3	22.2 ± 3.3	22.1 ± 3.1	22.1 ± 3.2	22.1 ± 3.3	22.2 ± 3.3
Salinity	34.2 ± 1.3	34.2 ± 1.2	34.2 ± 1.3	33.2 ± 1.7	33.1 ± 1.8	33.4 ± 1.7

*Sediment samples and water properties were measured at increasing distance from storm drains (0, 200, and 1,000 m away) in embayments and channels. Significant differences (p ≤ 0.05) of water and sediment properties among all sites are indicated with different lowercase letters. Parameters included water quality measurements (temperature, salinity), sediment metals (Co, Cr, Cu, Ni, and Zn), sediment properties [total nitrogen (TN), total organic carbon (TOC), and silt content (% <63 μm)]. For all results of the statistical analyses see Supplementary Table 6*.

These results are supported by the statistical analyses. In general, sediment properties differed with retention type and distance from storm drain (Table 1 and Supplementary Table 6). Specifically, in embayments we observed significantly lower concentrations of several metals such as Cu, Ni, and Zn in 1,000 m samples compared to those from 0 and 200 m samples. We also observed greater organic carbon and nitrogen content in embayment sediments 0 and 200 m away from the storm drain compared to 1,000 m sediments. In contrast, metal concentrations, total organic carbon and total nitrogen did not differ with distance from storm drains in channels and tended to be similar to samples collected at 1,000 m in embayments. The silt content (grain size) did not differ between channel and embayment sediments and was variable with distance within these retention types. Sediment properties also varied spatially (i.e., among locations within retention type), but showed almost no temporal variation (i.e., among sampling times). Water temperature and salinity were consistent among retention types and with distance from storm drains although there was some spatial variation among locations within retention type.

### Sediment Bacterial Community Composition

A total of 4,538,134 sequences with a mean of 47,272 (range: 29,919 to 69,706) sequences per sample were obtained after merging, quality filtering, denoising and removal of chimeric and non-bacterial sequences as well as singletons (Supplementary Table 3). Sequences were assigned to 15,954 bacterial zOTUs (Supplementary Table 2). A calculated rarefaction curve (Supplementary Figure 2A) as well as Michaelis-Menten Fit (Supplementary Table 3) confirmed that the sampling efforts of all samples were sufficient to represent the majority of the bacterial diversity (coverage: 77.4%). Species accumulation curves further indicated that more than 94% of all zOTUs (maximum number of zOTUs calculated = 16,928) were recovered by the surveying effort (Supplementary Figure 2B).

**Figure 2:**
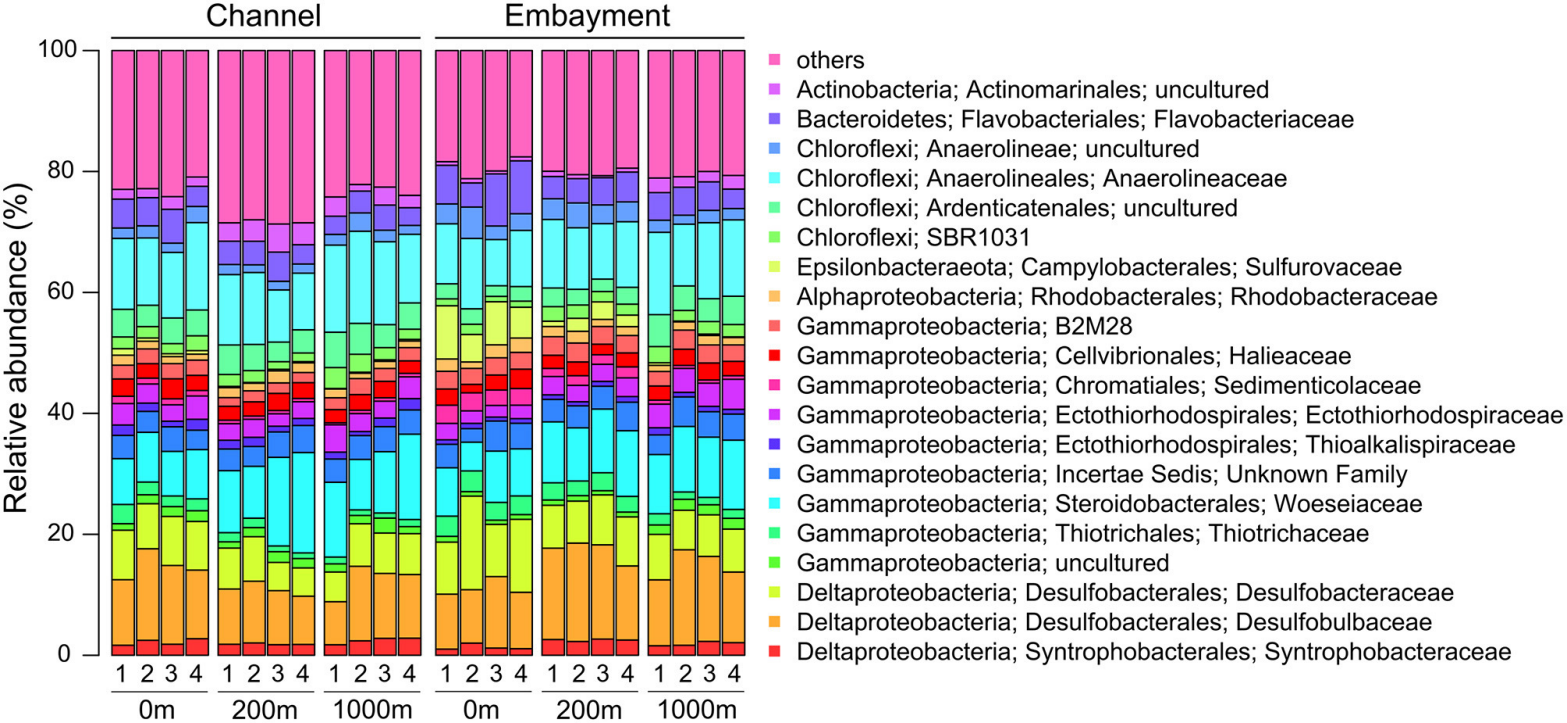
Abundant bacterial families in embayment and channel sediments as determined by 16S rRNA gene amplicon sequencing. Only families with an average abundance >1% in the entire data set are shown. 1, February; 2, March; 3, April; 4, May.

Six phyla dominated (>1% of all sequences across all samples) the entire bacterial community and accounted for more than 97% of all sequences analysed in this study (Supplementary Table 2). Approximately 60% of sequences across all samples were affiliated to *Proteobacteria* with *Gammaproteobacteria* (32.02%) as the predominant class, followed by *Deltaproteobacteria* (24.02%) and *Alphaproteobacteria* (3.39%). The other abundant phyla observed in this study were *Chloroflexi* (21.70%), *Bacteroidetes* (7.40%), *Actinobacteria* (3.22%), *Acidobacteria* (2.95%), and *Epsilonbacteraeota* (1.77%). The five dominant bacterial families were *Desulfobulbaceae* (11.58%), *Anaerolineaceae* (11.39%), *Woeseiaceae* (9.90%), *Desulfobacteraceae* (7.66%), and *Flavobacteriaceae* (4.58%; Figure 2).

### Bacterial Communities Differ in Relation to Estuarine Hydrology and Proximity to Storm Drain

**Figure 3:**
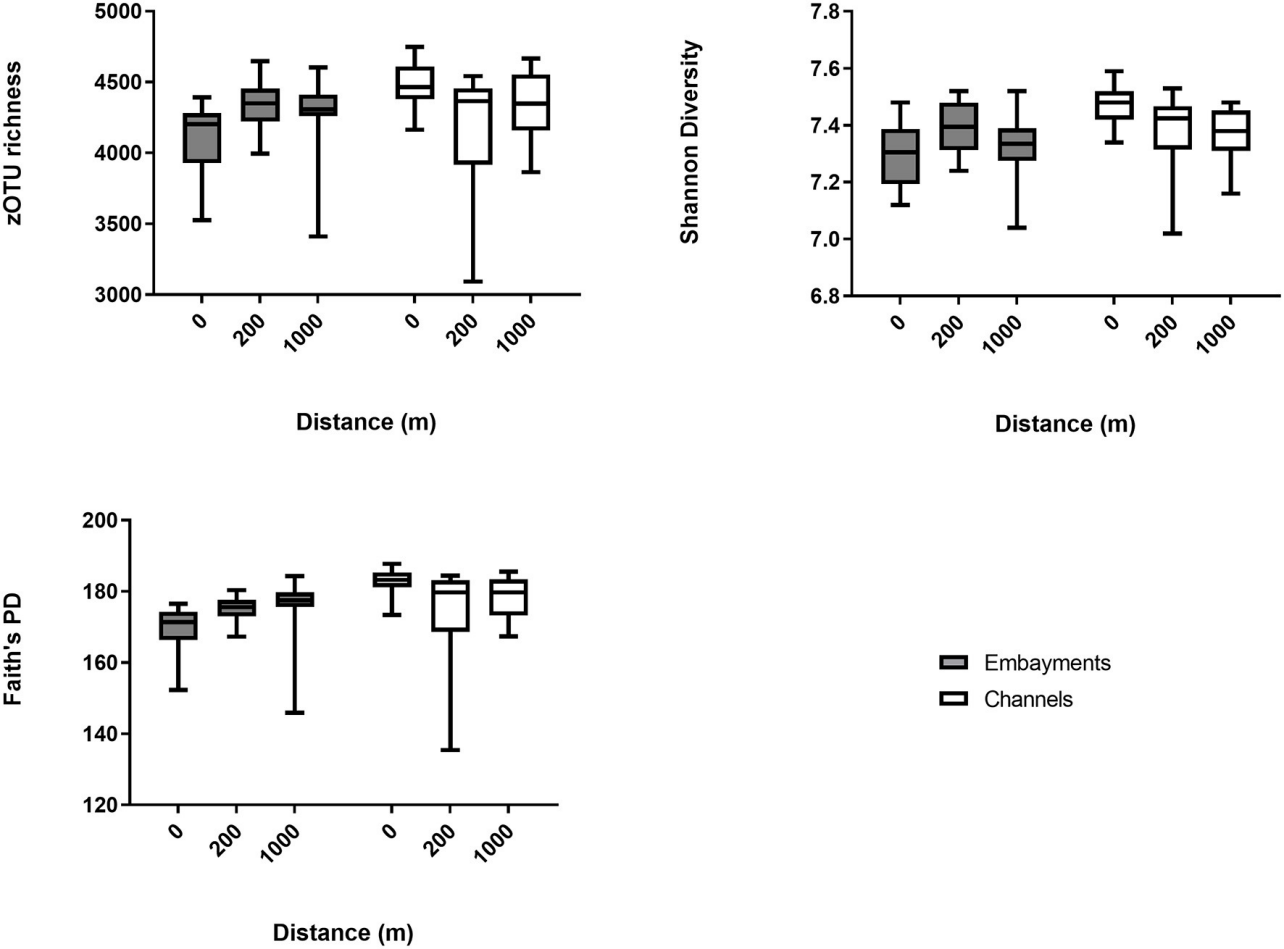
Alpha diversity measures (mean ± min/max) for benthic bacterial communities. Bacterial diversity was measured at increasing distance from storm drains (0, 200, and 1,000 m away) in embayments and channels. For all results of the statistical analyses (see Table 2).

We used linear mixed-effects models and pairwise comparisons to investigate the hypothesis that bacterial communities would differ between channels and embayments and would differ with proximity to storm drain only in embayments. Bacterial diversity, richness and Faith's PD varied as a result of the interactive effect of retention type and proximity to storm drain, and there was also significant spatial (among locations) and temporal (among sampling times) variation (Figure 3 and Supplementary Table 6). OTU richness, diversity and Faith's PD tended to be lowest in the 0 m samples from embayments compared to 200 and 1,000 m away from the storm drain (Figure 3 and Table 2). Similarly, bacterial diversity tended to be lower in 0 m samples of embayments compared to 0 m samples of channels, although this was not significant (Figure 3 and Table 2). In contrast, sediments sampled in channels did not differ in diversity with distance from storm drains (Figure 3 and Table 2). Sediments for microbial analyses were collected from the surface (<2 mm depth) to reflect exposure to changes in both the dynamic water column and more stable sediments. This may be why the microbial communities varied over time, but the sediment properties were generally constant.

**Table 2:** Statistical results of the pairwise comparisons investigating differences in bacterial (a) richness, (b) diversity and (c) Faith's PD.

**Comparison**	**Richness**		**Diversity**		**Faith's PD**	
	***t*-value**	***p*-value**	***t*-value**	***p*-value**	***t*-value**	***p*-value**
Embayment 0 m vs. Channel 0 m	2.16	0.1336	3.27	0.0343	2.70	0.0827
Embayment 0 m vs. Embayment 200 m	−3.10	**0.0028**	−2.83	**0.0060**	−2.51	0.0145
Embayment 0 m vs. Embayment 1,000 m	−2.49	0.0151	−1.02	0.3102	−2.91	**0.0049**
Embayment 0 m vs. Channel 200 m	−0.53	0.6410	−1.61	0.1887	−1.10	0.3605
Embayment 0 m vs. Channel 1,000 m	−1.34	0.2844	−1.44	0.2294	−1.63	0.2108
Embayment 200 m vs. Embayment 1,000 m	0.61	0.5444	1.81	0.0744	−0.40	0.6918
Embayment 200 m vs. Channel 0 m	0.83	0.4769	1.47	0.2202	1.55	0.2294
Embayment 200 m vs. Channel 200 m	−0.81	0.4875	−0.19	0.8587	−0.05	0.9603
Embayment 200 m vs. Channel 1,000 m	−0.01	0.9908	0.36	0.7375	−0.48	0.6668
Embayment 1,000 m vs. Channel 0 m	1.09	0.3667	2.62	0.0632	1.36	0.2752
Embayment 1,000 m vs. Channel 200 m	−0.54	0.6294	0.96	0.3960	−0.24	0.8293
Embayment 1,000 m vs. Channel 1,000 m	0.27	0.8044	0.79	0.4780	0.30	0.7871
Channel 0 m vs. Channel 200 m	3.81	**0.0003**	2.62	0.0108	3.48	**0.0009**
Channel 0 m vs. Channel 1,000 m	1.90	0.0616	2.89	**0.0051**	2.32	**0.0233**
Channel 200 m vs. Channel 1,000 m	−1.91	0.0606	0.27	0.7890	−1.17	0.2478

*Bacterial communities were sampled at increasing distance from storm drains (0, 200, and 1,000 m away) in embayments and channels. Significant differences (p ≤ 0.01) are indicated in bold*.

**Figure 4:**
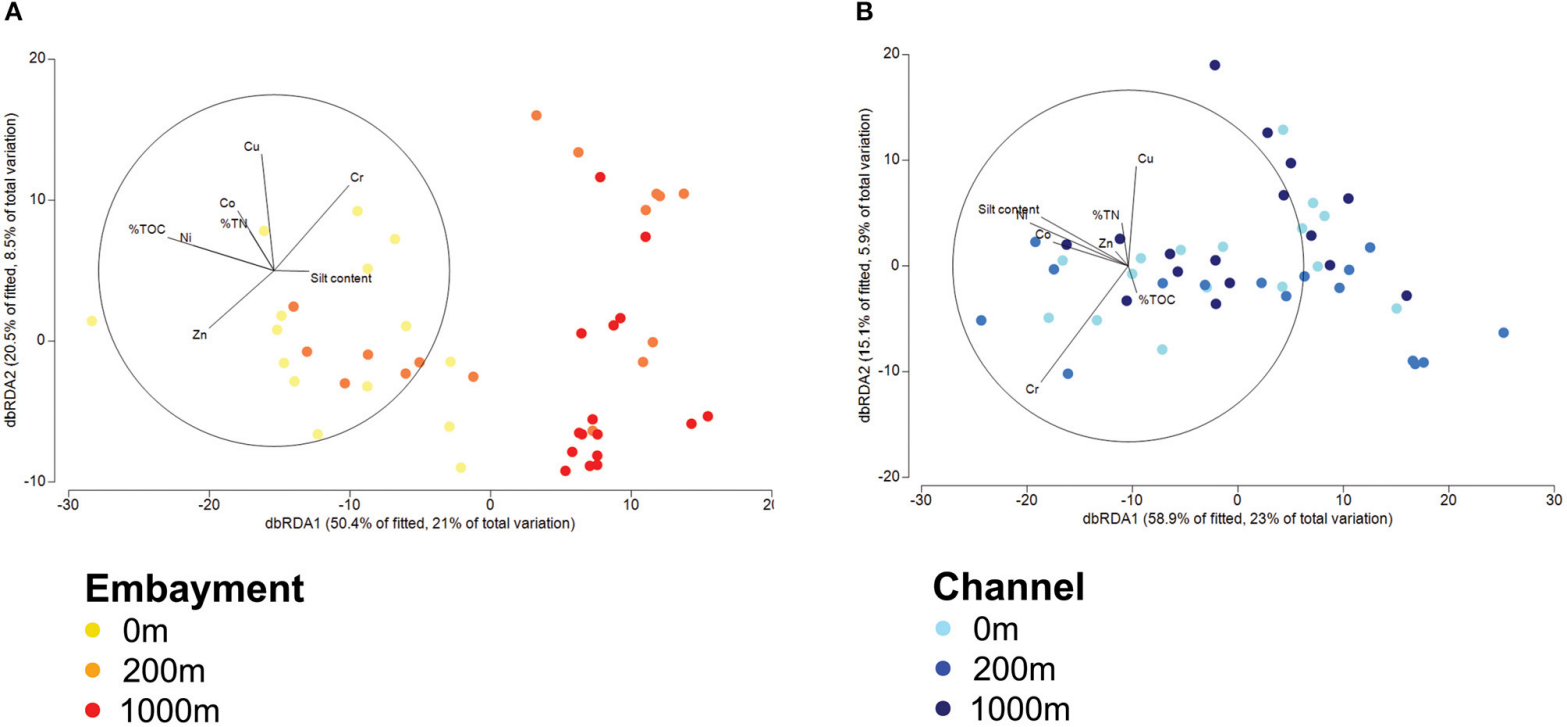
Distance-based redundancy analysis (dbRDA) of bacterial communities in embayments **(A)** and channels **(B)** with regard to the distance from storm drains fitted to predictor variables. Ordination is colour-coded by site (proximity to storm drain) and retention type. Blue symbols are channel locations and orange symbols are embayment locations. The gradient in colour from lighter to darker represents the distances 0, 200, 1,000 m from a storm drain. Lengths of vector overlays indicate the relative influences of fitted predictor variables.

Several predominant families differed with distance from stormdrains, with the strongest patterns occurring in embayments (Supplementary Figure 3 and Supplementary Table 7). *Flavobacteriaceae, Sulfurovaceae, Desulfobacteraceae, Sedimenticolaceae*, and *Thiotrichaceae* were most abundant next to storm drains and decreased in relative abundance with distance from the storm drain. The opposite trend was observed for *Desulfobulbaceae, Woeseiaceae, Actinomarinales, Ardenticatenales*, SBR1031*, Syntrophobacteraceae*, B2M28*, Ectothiorhodospiraceae* and *Thioalkalispiraceae*. In contrast, the relative abundances of the *Anaerolineaceae, Rhodobacteraceae*, and *Halieaceae*, were similar among retention types. Hence, we analysed the influence of proximity to storm drain and retention type as well as their interaction on bacterial community composition by permutational multivariate analysis of variance (PERMANOVA) and dbRDA analyses based on Bray-Curtis dissimilarities (Figure 4 and Supplementary Table 6). We observed a clear separation of sediment bacterial communities by proximity to storm drain, which was more pronounced for embayment than channel communities (*p* > 0.001, Figure 4). This was detected even accounting for significant spatial (among locations within retention type) and temporal (among sampling times) variation in bacterial community composition (Supplementary Table 6).

### Bacterial Communities in Channels and Embayments Are Related to Different Environmental Variables

To identify the potential drivers of bacterial community composition in channels and embayments, we performed separate statistical analyses for each retention type. Fitting the environmental variables to the bacterial zOTUs with dbRDA revealed metal concentrations, total nitrogen, total organic carbon and silt content in the sediments explained 42% of the variation in bacterial communities in embayments (Figure 4A, Table 3, and Supplementary Table 8) and 39% in channels (Figure 4B, Table 3, and Supplementary Table 8). Fewer environmental properties measured in this study were significant predictors of bacterial community composition in channels compared to embayments (Figure 4, Table 3, and Supplementary Table 8). Notably, several important sediment properties (Zn and total nitrogen) were only significant predictors of bacterial community composition in embayments.

**Table 3:** Statistical results of the PERMANOVA investigating potential drivers of benthic bacterial community composition.

	**Channels**		**Embayments**	
	***Pseudo-F***	***p-*value**	***Pseudo-F***	***p-*value**
Distance (m)	1.86	**0.0004**	2.31	**0.0001**
Al (mg/kg)	8.25	**0.0001**	4.93	**0.0001**
As (mg/kg)	3.00	**0.0002**	5.26	**0.0001**
Cd (mg/kg)	4.31	**0.0001**	8.77	**0.0001**
Co (mg/kg)	2.72	**0.001**	2.24	**0.0002**
Cr (mg/kg)	4.04	**0.0001**	2.29	**0.0001**
Cu (mg/kg)	1.30	0.1303	3.98	**0.0001**
Fe (mg/kg)	1.40	0.078	1.94	**0.0012**
Mn (mg/kg)	1.88	**0.0087**	2.24	**0.0002**
Ni (mg/kg)	1.26	0.1529	1.97	**0.0017**
Pb (mg/kg)	1.72	**0.0208**	2.12	**0.0002**
Zn (mg/kg)	1.19	0.2081	2.57	**0.0001**
TN (%)	1.51	0.0597	3.05	**0.0001**
TOC (%)	1.98	**0.0054**	1.24	0.1397
Silt content (% <63 μm)	3.25	**0.0002**	1.08	0.3239

*Bacterial community composition was measured at increasing distance from storm drains (0, 200, and 1,000 m away) in embayments and channels. Significant differences (p ≤ 0.05) along the distance gradient in channels and embayments are indicated in bold*.

### Active Bacterial Community in Embayments Differed With Proximity to Storm Drain

**Figure 5:**
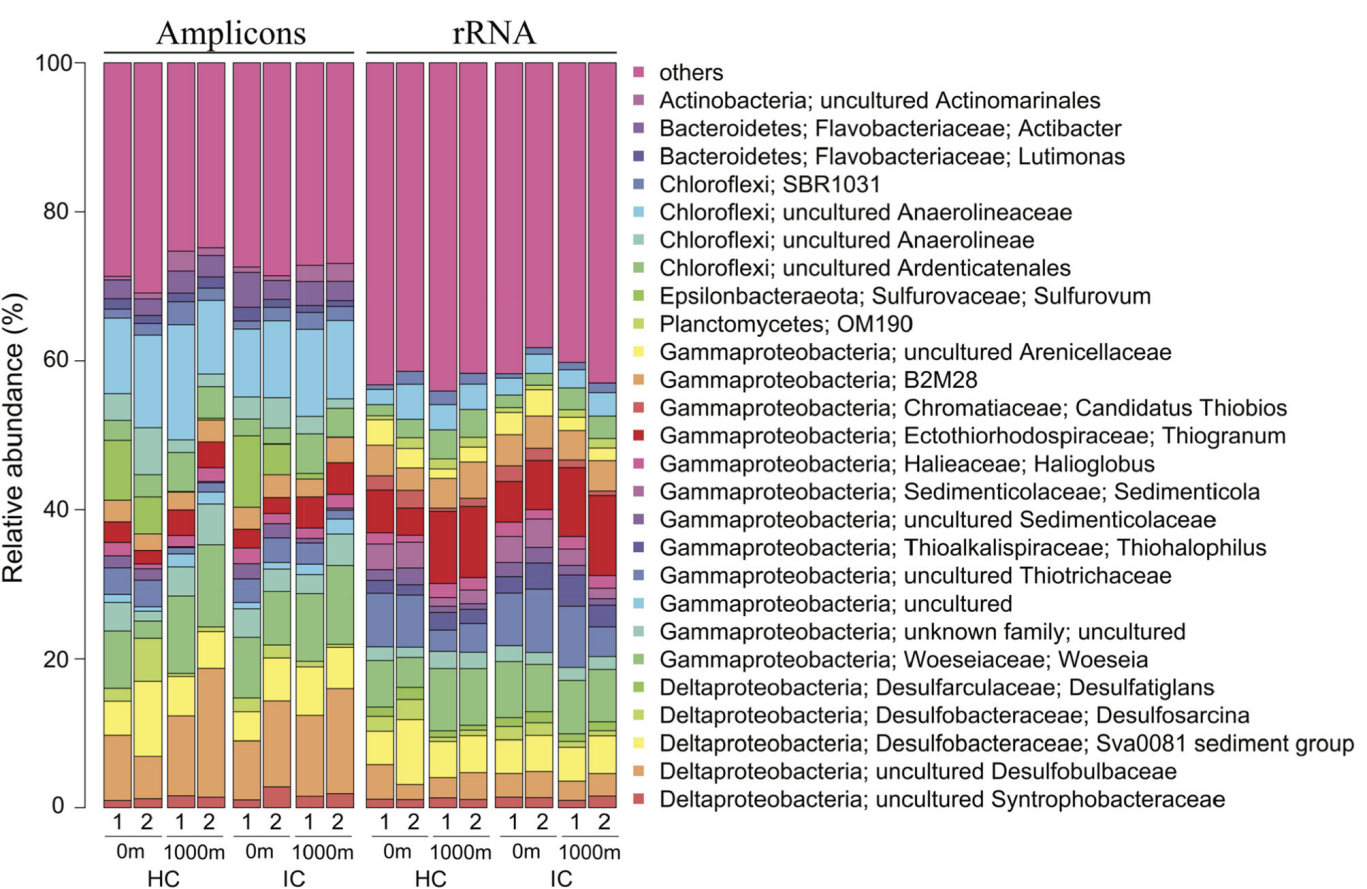
Abundant bacterial genera in embayments. Only genera with an average abundance > 1% in the 16S rRNA gene amplicon sequencing (entire) or in the rRNA (active) dataset, respectively, are shown. Entire and active bacterial communities were assessed by barcoding and mapping of rRNA reads on denoised unique sequences, respectively. HC, Hen and Chicken Bay; IC, Iron Cove; 1, February; 2, March.

We analysed samples collected close to and far from storm drains in two locations (Hen and Chicken Bay and Iron Cove) over 2 months (February and March 2014) by metatranscriptomics to test for differences in the community composition of the active bacteria among sites. Active bacterial communities across all samples were dominated by a few bacterial genera, such as *Thiogranum* (7.60%), *Woeseia* (6.82%), the Sva0081 sediment group (5.23%) and the B2M28 group (4.06%) (Figure 5 and Supplementary Table 4). At the DNA level, uncultured bacteria of the *Desulfobulbaceae* (10.90%) and *Anaerolineaceae* (11.20%) were predominant. Other abundant genera were, for example, *Woeseia* (8.30%), uncultured members of the Sva0081 sediment group (5.84%) and *Thiogranum* (3.08%). Several bacterial genera including *Lutimonas, Actibacter*, or *Sulfurovum* were abundant (>1% of the abundance in the DNA dataset) in the entire bacterial community, but rare (<1% of the abundance in the RNA dataset) in the active bacterial community. In contrast, *Sedimenticola, Desulfosarcina, Desulfatiglans*, and members of the OM190 group were highly abundant in the active, but not in the entire bacterial community.

**Figure 6:**
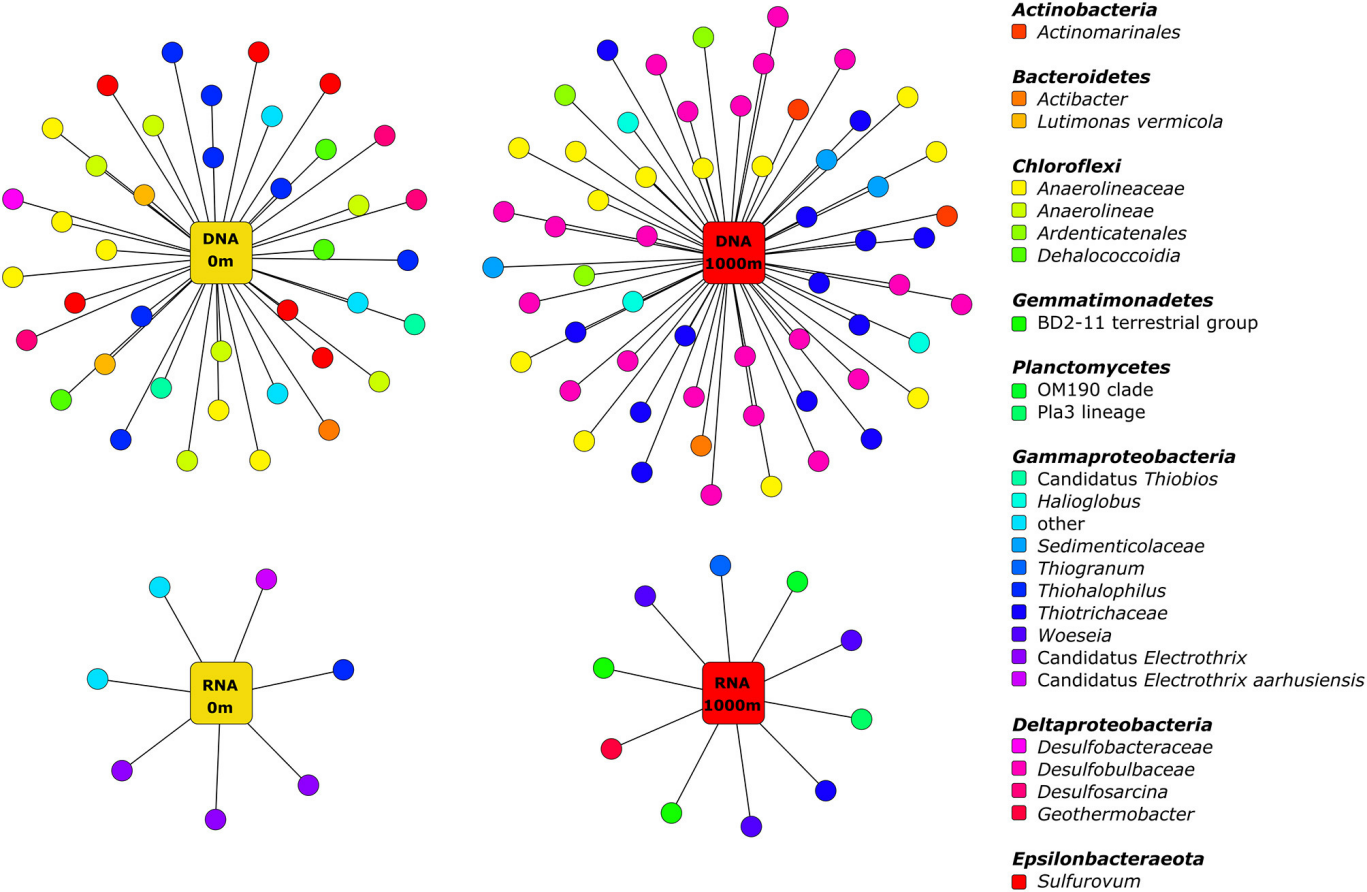
Bipartite association network of significantly associated bacterial taxa close to and far from storm strains in embayment sediments. Only uniquely associated taxa from entire (DNA) and active (RNA) bacterial communities are shown. For further information see Supplementary Table 5.

Several of the predominant bacterial genera also differed in their relative abundances with distance from storm drains (Supplementary Figures 4, 5 and Supplementary Table 9). Consequently, we performed an indicator species analysis to identify bacterial zOTUs significantly associated with 0 and 1,000 m in embayments (Figure 6 and Supplementary Table 5). The number of bacterial zOTUs that differed with proximity to a stormdrain was higher in the entire (DNA) community compared to the active (RNA) bacterial community. Several bacteria including two zOTUs belonging to *Lutimonas vermicola* and three zOTUs of the genus *Sulfurovum* were significantly associated with 0 m samples of embayments, whereas others including zOTUs belonging to the genera *Halioglobus, Woeseia* and *Thiogranum* were uniquely associated with 1,000 m samples. In the rRNA dataset, three zOTUs belonging to Candidatus *Electrothrix*, one zOTU affiliated with Candidatus *Electrothrix aarhusiensis* as well as uncultured members of the *Sedimenticolaceae* and *Thiotrichaceae* were significantly associated with 0 m samples. Two zOTUs belonging to BD2-11 terrestrial group, OM190, the Pla3 lineage, *Geothermobacter* and *Thiohalophilus* showed unique associations with 1,000 m samples.

**Figure 7:**
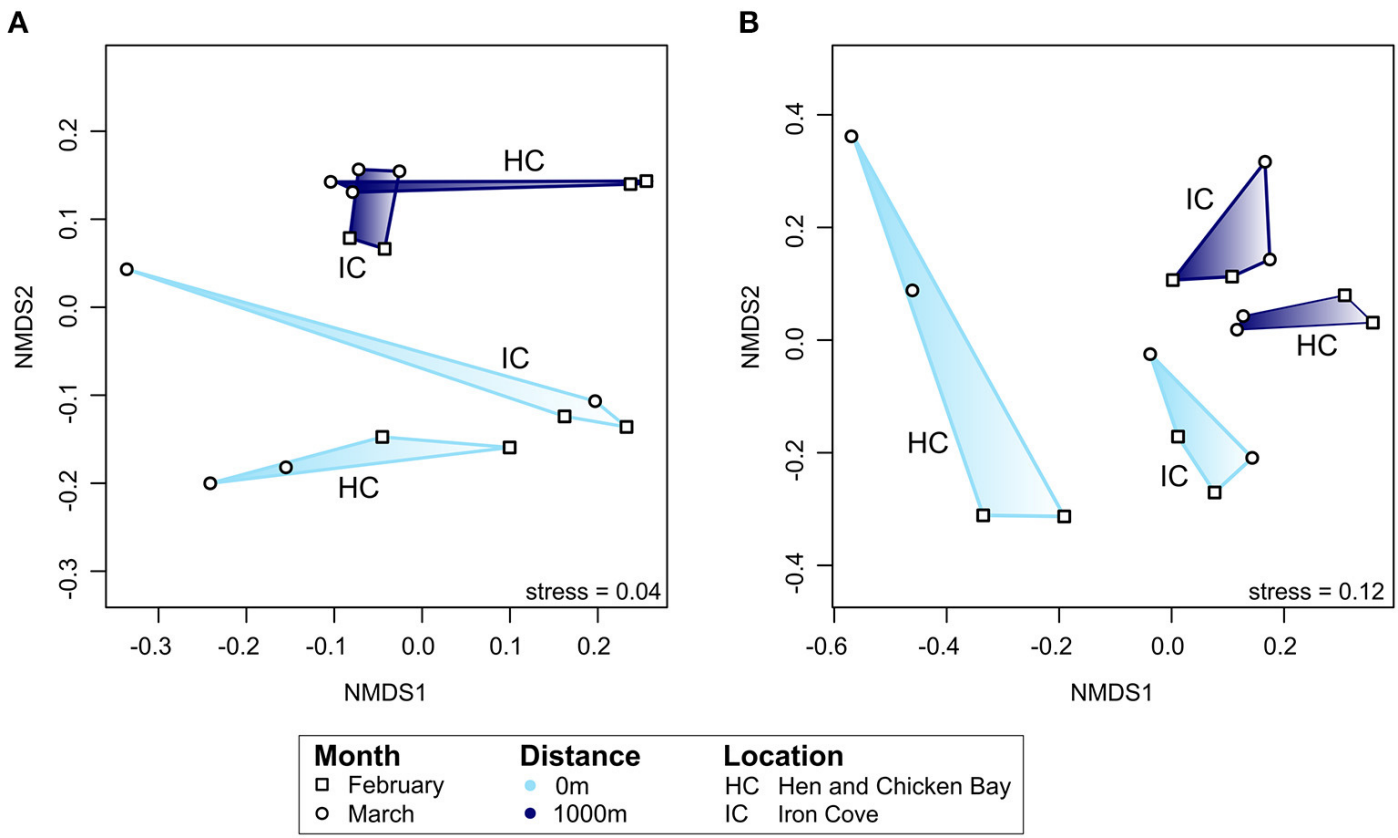
Ordination analyses of the composition of the active bacterial community [rRNA; **(A)**] and assembled non-rRNA transcripts **(B)**. Only transcripts with known functional annotation were included in the analysis. Ordination is based on Bray-Curtis dissimilarities between samples and is colour-coded by distance from storm drain. Different symbols represent the two sampling months, February or March. Note that the nMDS axes have different scales for each ordination. The ordination plot for all (total) non-rRNA transcripts and those transcripts without known functional annotation can be found as Supplementary Figure 6. Data included in Supplementary Table 4. IC, Iron Cove; HC, Hen and Chicken Bay.

The active bacterial community differed significantly with proximity to storm drain and this was consistent among sampling times (Figure 7A). Specifically, the bacterial community composition differed significantly between sites close to and far from storm drains in February (*p* = 0.03, *R*^2^ = 38.6%) and March (*p* = 0.03, *R*^2^ = 38.6%) and this pattern was consistent among locations (Figure 7A).

We further expected that gene expression of bacterial communities in embayments would differ between sediments close to and far from storm drains. To investigate this, we performed ordination analyses based on Bray-Curtis dissimilarities for assembled non-rRNA transcripts with known functional annotation (Figure 7B). We found a clear separation by proximity to storm drain and sampling month. Similar results were obtained when investigating all assembled transcripts as well as transcripts without known functional annotation. Statistical analysis by PERMANOVA revealed that the compositon of assembled transcripts with known functional annotation differed with distance from storm drains (*p* = 0.001, *R*^2^ = 7.06%) and sampling time (*p* = 0.04, *R*^2^ = 7.20%). In addition, these transcripts differed significantly among sites in February (*p* = 0.03, *R*^2^ = 16.5%) and March (*p* = 0.04, *R*^2^ = 16.9%).

**Figure 8:**
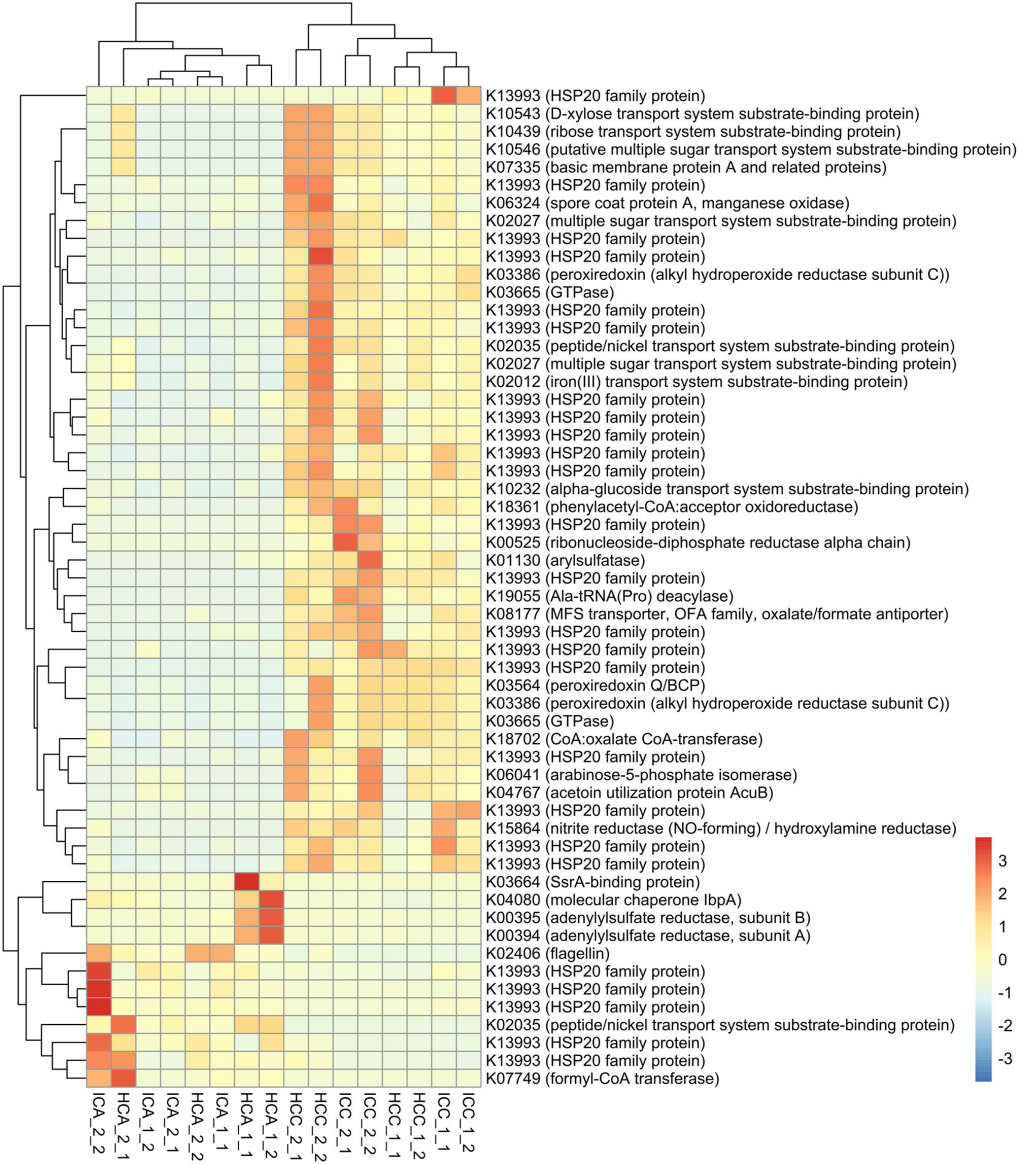
Heatmap showing transcripts which were differentially expressed between 0 and 1,000 m samples of embayments. IC, Iron Cove; HC, Hen and Chicken Bay; A, 0 m; C, 1,000 m; 1_1, February Replicate 1; 1_2, February Replicate 2; 2_1, March Replicate 1; 2_2, March Replicate 2. The colour scale refers to log2 fold change normalised to the mean in each sample.

We additionally identified 56 bacterial transcripts that were differentially expressed (*p* ≤ 0.001) between 0 and 1,000 m samples (Figure 8). The majority of these transcripts belonged to the HSP20 family (Heat shock proteins). Most transcripts encoding for the HSP20 family as well as multiple transcripts including those encoding for iron(III) transport system substrate-binding protein (K02012), nitrite reductase (NO-forming)/hydroxylamine reductase (K15864) and spore coat protein A (manganese oxidase; K06324) were expressed more at 1,000 m sites than other sites closer to stormwater outlets. By contrast, higher expressions of transcripts encoding for formyl-CoA transferase (K07749]) were detected in embayments close to storm drains. The transcripts for peptide/nickel transport system substrate-binding protein and for adenylylsulfate reductase, subunit A (K00394) and subunit B (K00395), were expressed significantly more at sites close to the storm drain at Hen and Chicken Bay, while a significantly higher expression of transcripts encoding for the arylsulfatase (K01130) were detected in Iron Cove samples collected at 1,000 m.

## Discussion

Over the past decade, the growing problem of contamination in marine systems, particularly urban estuaries, has resulted in an increase in research focused on understanding the impact of anthropogenic stressors such as metals or excess nutrients on bacterial communities (e.g., Liu et al., 2014; Beale et al., 2018; Birrer et al., 2018; Su et al., 2018). To date, most studies have focused on changes in diversity and community composition. Consequently, the functional responses of these communities to contaminant stressors are poorly understood. Here, we showed that alpha diversity measures and the composition of bacterial communities were affected by the hydrology that influences retention of water and sediment contaminants. We further demonstrated that bacterial communities in embayments differed more among the distance gradient from storm drains than in channels. These findings support our hypotheses that bacterial communities in channels and embayment systems respond differently to proximity to stormwater inputs. Further we found that the composition and functions of the active bacterial community in embayments differed between sites close to and far from storm drains. In addition, we found greater expression of several genes involved in bacterial stress response at sites further away from than close to storm drains. These findings suggest that bacterial communities in sites close to storm drains may be better adapted to anthropogenic stressors.

### Relationships Between Estuarine Hydrology and Benthic Bacterial Diversity

Ecological monitoring studies in soft sediments have traditionally used diversity metrics as indicators of anthropogenic disturbance with the expectation that effects of contaminants might include decreased richness and evenness of communities (Johnston and Roberts, 2009). Our previous work has established that bacteria are as sensitive, if not more sensitive than archaea and eukaryotic microbes to the stressors examined in this study (Sun et al., 2012, 2013; Birrer et al., 2018, 2019). Here we showed that alpha diversity measures and the overall composition of bacterial communities were affected by retention type (hydrology). Specifically, bacterial diversity tended to be lower in the embayments compared to channels and was lowest in proximity to storm drains. This pattern of reduced diversity occurred where contaminant concentrations including Cu, Ni, Pb, and Zn were highest, and sediments were also highly enriched with organic carbon and nitrogen. Thus, contaminants accumulating in the sediments adjacent to stormdrains in poorly flushed embayments may be contributing to loss of sensitive species and increased dominance of more tolerant taxa (Johnston and Roberts, 2009). Some specific groups of bacteria also differed with respect to retention type. We found that *Desulfobacteraceae* and *Desulfobulbaceae* were more abundant in embayments, with *Desulfobacteraceae* most abundant next to storm drains. Members of the families Desulfobacteraceae and Desulfobulbaceae are well known sulphate-reducing bacteria (Leloup et al., 2005; Gittel et al., 2008). Sulphate-reducing bacteria display a certain degree of metal tolerance as a secondary outcome of their metabolism (Valls and De Lorenzo, 2002). Our findings highlight the abundance of sulphate-reducing bacteria in embayments in Sydney Harbour and how their distributions might be related to hydrological conditions in estuaries. While Sydney Harbour is a large and complex natural harbour, future studies that include multiple estuaries under a range of rainfall conditions would be useful to investigate the broader applicability of our findings.

### Legacy Contamination Near Storm Drains in Embayments Linked to Altered Bacterial Community Composition and Functioning

Our analyses also revealed that benthic bacterial communities were related to various natural and anthropogenic properties such as metals and nutrients in both channels and embayments. A study by Yan et al. (2018) observed that TN and TC of the sediment samples significantly affected bacterial communities in mudflat sediments from the Dongtan wetland of Chongming Island. Partly in line with our and the above-mentioned study, TN had no effect on benthic bacterial communities along the Pearl Estuary (China), while TOC significantly correlated with these communities (Liu et al., 2014). Interestingly, in our study metal concentrations better predicted bacterial community composition in embayments than channels. Specifically, bacteria from embayments but not in channels differed among the distance gradient from storm drains and we observed significantly lower concentrations of several metals and nutrients in embayment samples 1,000 m away from the storm drain compared to those from 0 and 200 m. This supports our hypothesis that bacterial communities in channel and embayment systems respond differently to proximity to legacy stormwater contamination, and is in agreement with previous observations that nutrients and metals accumulate close to input sources (Liebens, 2001; Birch and Rochford, 2010). Differences may be occurring because the bacterial community living in close proximity to storm drains in embayments has recruited under prevailing contaminant concentrations. Thus, high metal concentrations may have selected for those microorganisms more tolerant to metals as proposed by Gillan et al. (2005). Alternatively, the effect of metals on the bacterial community may be altered by the presence of excess nutrients in a so-called “antagonistic” interaction of multiple stressors. This might be supported by the higher total nitrogen and total organic carbon content found at 0 and 200 m in embayments compared to other embayment and channel sediments although only total nitrogen was a significant predictor of community composition. It should also be acknowledged that the microbial communities were sampled from the surface sediments (<2 mm depth) and thus variation may also reflect water or sediment properties that were not measured here such as light attenuation and pore water, respectively. However, experimental studies by the authors have identified direct causal impacts of metals and nutrients in sediments changing microbial communities (Birrer et al., 2018, 2019), and these stressor variables were statistically correlated with changes in this survey study and related studies (Sun et al., 2012, 2013; Dafforn et al., 2014).

In this study, the predominant genera between entire and active bacterial community differed significantly, which is in line with previous studies (Campbell and Kirchman, 2013; Wemheuer et al., 2017). While we cannot directly compare active and entire bacterial communities, as we used different approaches for the assessment, we observed that the uniquely associated zOTUs based on RNA-Seq analysis differed from those based on 16S rRNA gene sequencing. Nonetheless, consistent with our hypotheses and patterns in the entire bacterial community, the composition and functions of the active bacterial community in embayments differ between sites close to and far from storm drains. Such shifts may be explained by differences in metal and nutrient concentrations observed in the present study. Similarly to our study, significant changes in bacterial community composition and predicted functions, such as altered activity of genes involved in the nitrogen cycle as a response to anthropogenic activities, were recorded in a recent study on bacterial communities in surface sediments of Hangzhou Bay (Su et al., 2018). Dell'anno et al. (2003) showed in their study on the impact of metal contaminants on bacterial activities in coastal marine sediments that bacterial metabolism and turnover was inhibited by high metal concentrations. Our results together with previous studies (Wemheuer et al., 2015; Su et al., 2018) suggest that shifts in bacterial community composition result in altered community function.

Given the significant contamination found at sites close to storm drains, we also expected that the communities present would be highly stressed. Instead we found that several genes involved in bacterial stress response were more expressed at sites further away from storm drains. Further analysis revealed that the majority of differentially expressed transcripts belonged to the Hsp20 family. Heat shock proteins are involved in a universal molecular stress response and play an important role for cell growth and viability (Jung and Lee, 2012; Li, 2017). Previous studies showed that Hsp20 is involved in cellular defence under environmental stress conditions. In a study on the marine ciliate Euplotes crassu, many Hsps such as Hsp20 were up-regulated after metal exposure (Kim et al., 2018). Interestingly, these previous findings do not match our patterns in stress responses since metal concentrations tended to be higher near storm drains in embayments. However, this could be because the communities living in these degraded conditions are already tolerant to the contaminants present or less tolerant taxa have been subject to environmental filtering.

In addition to transcripts involved in stress response, we identified several other differentially expressed transcripts involved in the sulphur cycle such as the adenylylsulfate reductase (adenosine 5′-phosphosulfate [APS] reductase) or the arylsulfatase. The adenylylsulfate reductase plays an important role in catalysing APS to sulfite in the dissimilatory sulphate reduction pathway and thus represents a key enzyme in the energy metabolism of sulphate-reducing prokaryotes. The higher expression of adenylylsulfate reductase close to the storm drain in Hen and Chicken Bay may be related to higher organic content. In another study on arylsulfatase activity and arylsulfatase-producing bacteria in sediment samples collected from marine, estuarine and mangrove biotopes, higher substrate concentrations tended to inhibit the arylsulfatase activity (Chandramohan et al., 1974). This is supported by the present study, as a significantly higher expression of transcripts encoding for the arylsulfatase were detected in Iron Cove samples collected at 1,000 m where organic enrichment was lower.

In total, 96 and 16 sediment samples were analysed by 16S rRNA gene sequencing and RNA-Seq analysis, respectively. Calculated rarefaction and species accumulation curves confirmed that the library size was large enough to reflect the bacterial diversity in sediments investigated in the amplicon-based dataset. Nonetheless, the results of the current study are only valid for the proportion of the bacterial community covered by the surveying effort.

### Bacterial Indicators for Biomonitoring at Storm Drains

Biomonitoring indicators need to fulfil several objectives (Rainbow, 1995). Firstly, they should be sensitive to the stressor of interest (e.g., metals and/or nutrients) and need to provide ecologically relevant information. Secondly, biomonitoring indicators need to produce repeatable information (i.e., low variability in the results from different replicates of the same treatment) and be reproducible in other systems with different contaminant concentrations. Finally, biomonitoring indicators should be transferable from the scientific sector to monitoring programs for easy and cost-effective application (Bourlat et al., 2013). Through indicator analysis, we identified several zOTUs belonging to the important sulphur-oxidising bacterial genera *Thiogranum* or *Sulfurovum* (Inagaki et al., 2004), which were significantly associated with 1,000 or 0 m samples, respectively. Furthermore, several bacterial zOTUs belonging to Candidatus *Electrothrix* showed unique associations with sites close to storm drains. These cable bacteria are long, multicellular filaments, that can conduct electric currents over centimetre-scale distances (Trojan et al., 2016). Taxa associated with 1,000 m sites could indicate sensitivity to elevated metal and nutrient concentrations since these decreased with distance from storm drains. Likewise, taxa significantly associated with 0 m sites might be more tolerant to multiple anthropogenic stressors. These are initial patterns and further testing including experimental manipulation would be needed to determine if these are important bacterial indicators for contaminants in estuarine sediments and could be applied more broadly for biomonitoring.

## Conclusion

Our results indicate that the impact of environmental properties on benthic bacterial communities is largely determined by estuarine hydrology. Furthermore, higher nutrient and metal concentrations close to storm drains in embayments are important predictors of benthic bacterial community structure and function. These patterns were detectable over and above significant spatial and temporal variation suggesting wider applicability than just Sydney Harbour. Our results therefore contribute to a better understanding of benthic bacterial responses to different retention types and associated anthropogenic stressors and have implications for future management practices in highly urbanised estuaries. An appropriate management action might be to redirect stormwater discharge points into faster flowing waters where any associated contaminants will be diluted and flushed, thus lessening any localised impacts. Future studies should assess how benthic bacterial communities and their functions in estuaries are affected upon large storm events and additional studies in estuaries with varied morphology and flushing characteristics would lend weight to our conclusions.

## Data Availability Statement

Sequence data were deposited in the sequence read archive (Ghosh et al., 2006) of the National Center for Biotechnology Information (NCBI) under accession numbers SUB7398817 (amplicons) and SUB7403872 (metatranscriptomes). All other data can be found in the Supplementary Material.

## Author Contributions

SB, KD, PG, PSt, SS, JP, PSc, MD, and EJ contributed to the experimental design. SB and KD collected the field samples. SB, FW, and KD analysed the data and wrote the manuscript text. All authors edited the manuscript text.

## Funding

This research was funded by an ARC Linkage Grant (LP130100364) awarded to EJ, PSt, PG, MD, SS, and PSc. Fieldwork was conducted under NSW Department of Primary Industries permit number P13/0007e1.0.

## Conflict of Interest

The authors declare that the research was conducted in the absence of any commercial or financial relationships that could be construed as a potential conflict of interest.

## Publisher's Note

All claims expressed in this article are solely those of the authors and do not necessarily represent those of their affiliated organizations, or those of the publisher, the editors and the reviewers. Any product that may be evaluated in this article, or claim that may be made by its manufacturer, is not guaranteed or endorsed by the publisher.
