# Supplementary material for: Legacy Metal Contaminants and Excess Nutrients in Low Flow Estuarine Embayments Alter Composition and Function of Benthic Bacterial Communities
Source: Front Microbiol. 2021 Oct 8;12:661177. doi: 10.3389/fmicb.2021.661177 (PMC8531495; doi:10.3389/fmicb.2021.661177)
Supplement: Supplementary file 1 [file Data_Sheet_1.zip › Data Sheet 1.DOCX]

**Supplementary Materials**

**Tables**

**Table S1. a) Study design and environmental properties.** Samples coloured in red were selected for metatranscriptomic analysis. Water temperature and salinity were measured once. All other environmental properties were measured twice. Only the means are shown. **b) PCA Eigenvalues and Eigenvectors for environmental properties displayed in Supplementary Figure S1.** Variables (Temperature, Salinity, Co, Cr, Cu, Ni, Zn, %TN, %TOC, Silt content) were selected from a Draftsman Plot to account for co-linearity of variables (Al, As, Cd, Fe, Mn, Pb).

**Table S2. zOTU table for the entire bacterial community in sediments.** For further information on the Sample ID see Table S1.

**Table S3. Sequence characteristics (number of sequences) and alpha diversity values.** Alpha diversity values were calculated at the same surveying effort (number of sequences = 29,919). Every value was calculated 10 times in R. The average of all 10 iterations is provided.

**Table S4. zOTU and transcript tables for the active bacterial community in sediments.** For further information on the Sample ID see Table S1.

**Table S5. Significantly associated bacterial zOTUs with distance.** Only uniquely associated taxa from entire (DNA) and active (RNA) bacterial communities are shown

**Table S6. Statistical results for the overall effect of retention type and distance on environmental properties and alpha and beta diversity measures.** Data were analysed with sampling time and/or location (nested in retention type) as random effects and retention type as well as distance as fixed factors. Univariate measures were analysed with linear mixed models and significance levels for fixed factors are based on F-values, calculated by a type III analysis of variance with Satterthwaite approximation for degrees of freedom. For random factors, an ANOVA-like table with likelihood ratio test (LRT) statistics was generated. Multivariate measures were analysed with PERMANOVA and significance levels for fixed and random factors are based on Pseudo-F values, calculated with Type III sums of squares. Number of observations: 96; number of locations: 4, number of retention types: 2, number of sampling time: 4, number of distances: 3. ***: p < 0.001/**: p < 0.01/*: p < 0.05/n.s.: p > 0.05. Sampling time was the replicate in analyses for water temperature and salinity.

**Table S7. Statistical results for the overall effect of retention type and distance on abundant bacterial families.** Data were analysed with sampling time and/or location (nested in retention type) as random effects and retention type as well as distance as fixed factors. Univariate measures were analysed with linear mixed models and significance levels for fixed factors are based on F-values, calculated by a type III analysis of variance with Satterthwaite approximation for degrees of freedom. For random factors, an ANOVA-like table with likelihood ratio test (LRT) statistics was generated. Number of observations: 96; number of locations: 4, number of retention types: 2, number of sampling times: 4, number of distances: 3.

**Table S8. Results of the DISTLM analysis of bacterial community composition (zOTUs) against environmental predictors.**

**Table S9. Statistical results for the overall effect of retention type and distance on abundant bacterial genera.** Data were analysed with sampling time and/or location (nested in retention type) as random effects and retention type as well as distance as fixed factors. Univariate measures were analysed with linear mixed models and significance levels for fixed factors are based on F-values, calculated by a type III analysis of variance with Satterthwaite approximation for degrees of freedom. For random factors, an ANOVA-like table with likelihood ratio test (LRT) statistics was generated. Number of observations: 16; number of locations: 2, number of distances: 2.

**Figures**

**
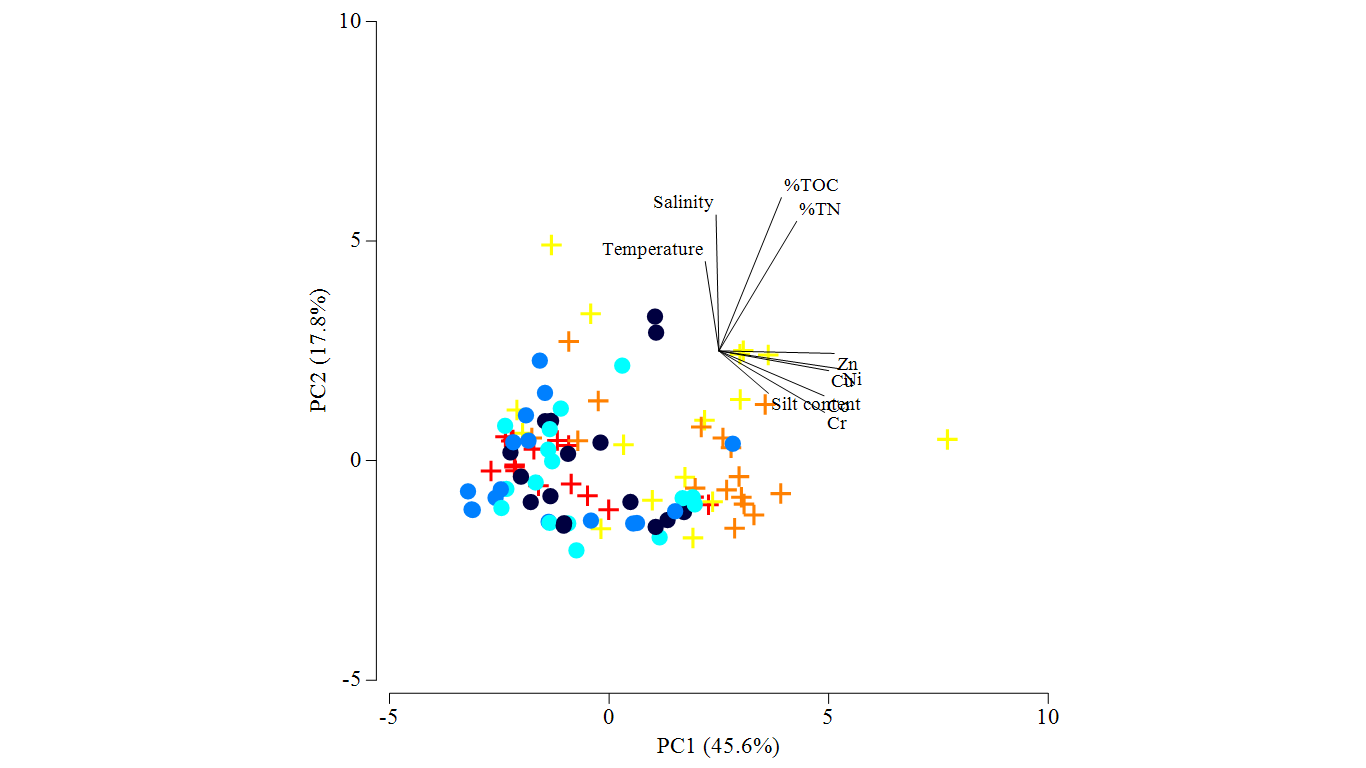
**

**Figure S1: Principal Components Analysis ordination for environmental characteristics from Sydney Harbour, Australia.** For further explanation on environmental data see Supplementary Table S1. Blue symbols are channel locations and orange symbols are embayment locations. The gradient in colour from lighter to darker represents the distances 0 m, 200 m, 1000 m from a storm drain.

**
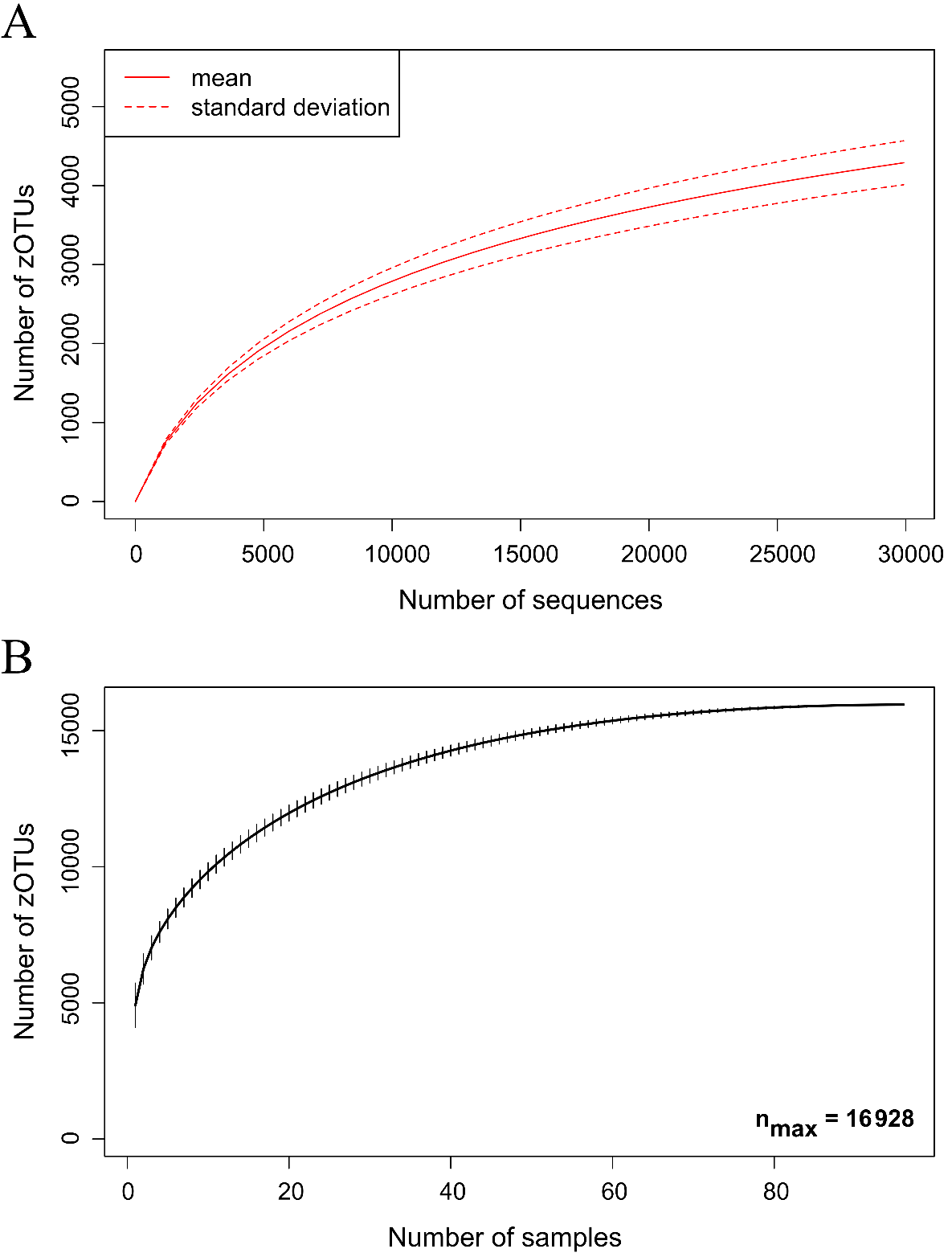
**

**Figure S2: Rarefaction (A) and species accumulation curves (B) calculated for the entire bacterial community**. The maximal number of zOTUs (n_max_) was calculated based on Michaelis Menten.

**
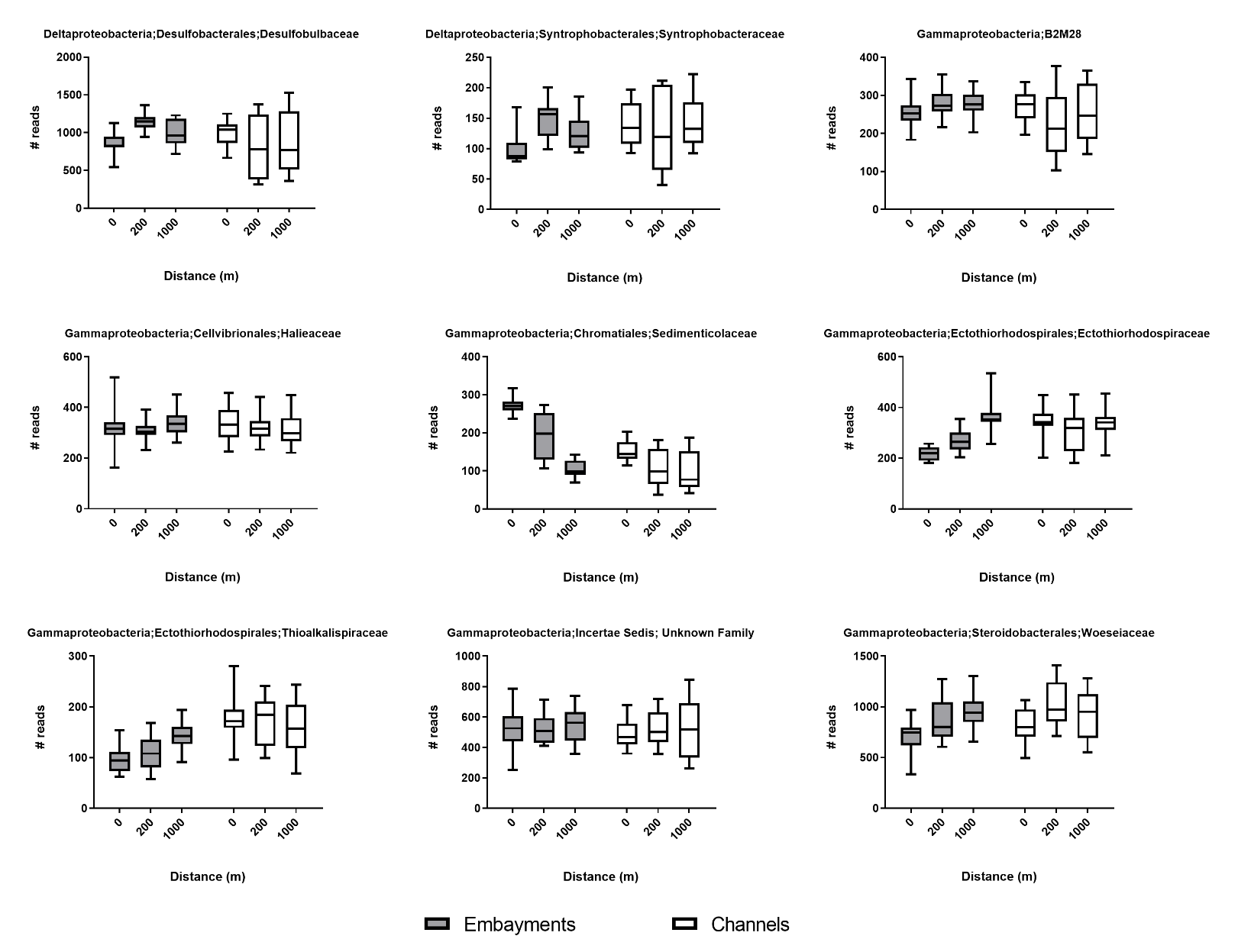
**

**Figure S3: Boxplots showing mean (min/max) of abundant bacterial families in embayment and channel sediments as determined by 16S rRNA gene amplicon sequencing.** Only families with an average abundance > 1% in the entire data set are shown.

**
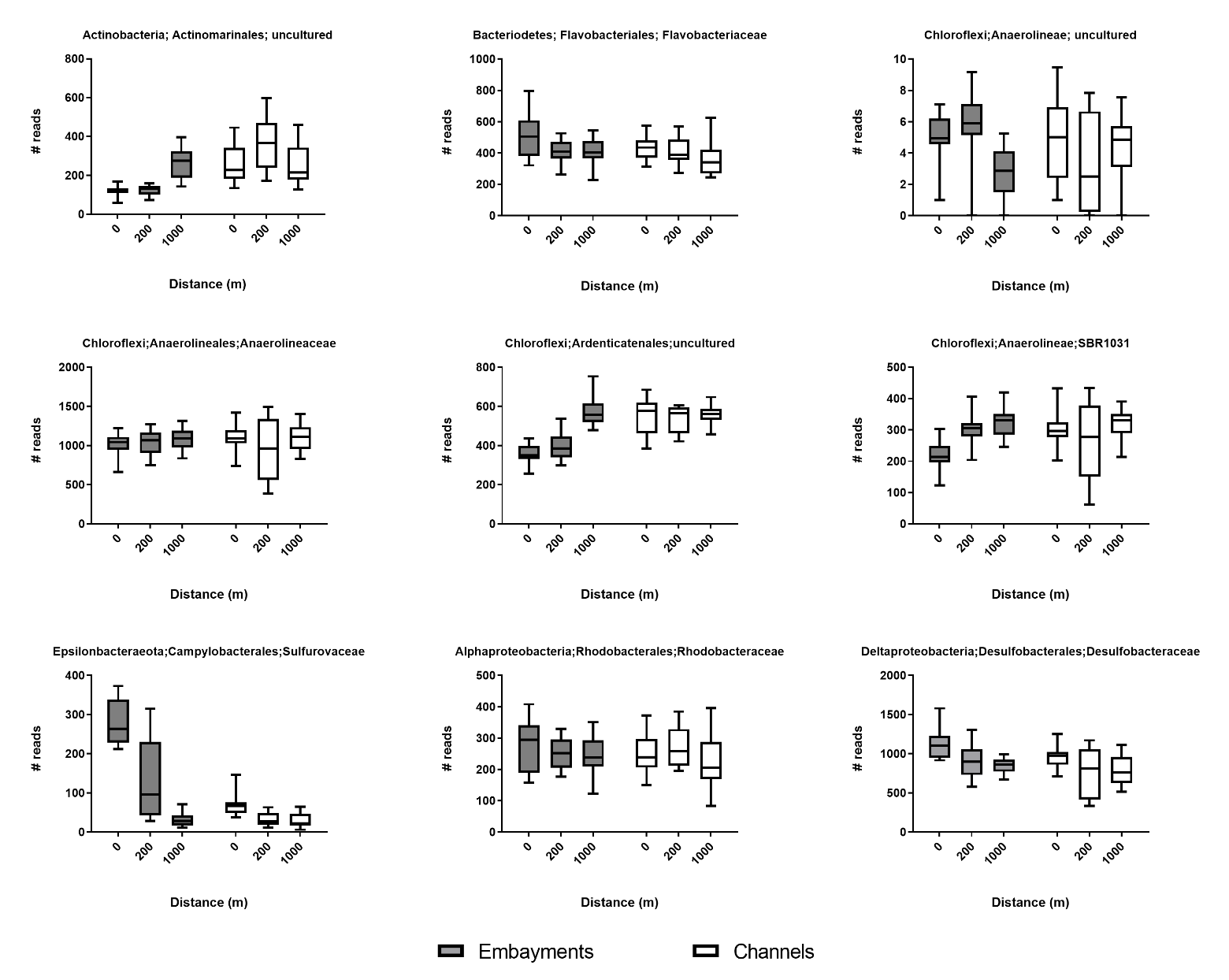
**

**Figure S3 cont.: Boxplots showing mean (min/max) of abundant bacterial families in embayment and channel sediments as determined by 16S rRNA gene amplicon sequencing.** Only families with an average abundance > 1% in the entire data set are shown.

**
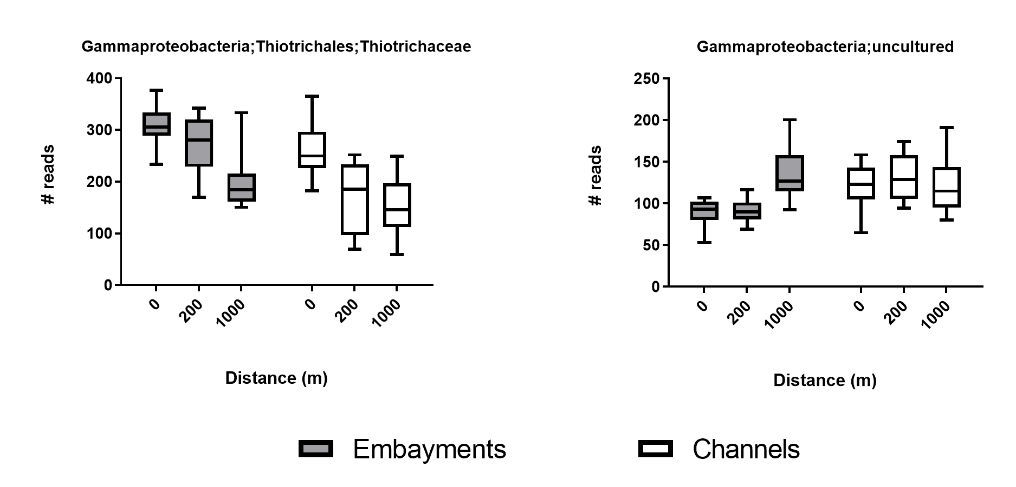
**

**Figure S3 cont.: Boxplots showing mean (min/max) of abundant bacterial families in embayment and channel sediments as determined by 16S rRNA gene amplicon sequencing.** Only families with an average abundance > 1% in the entire data set are shown.

**
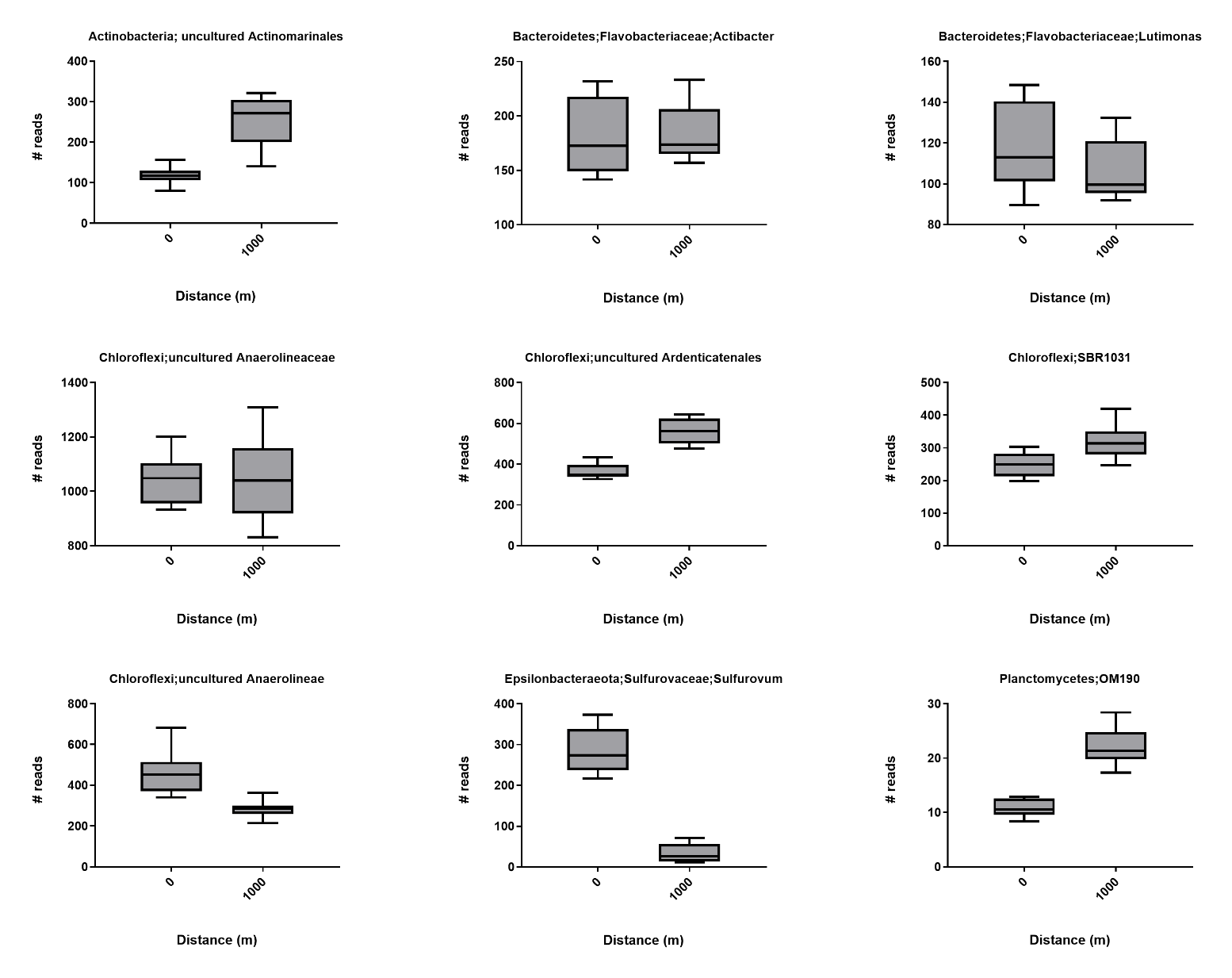
**

**Figure S4: Boxplots showing mean (min/max) of** **abundant bacterial genera in embayments.** Only genera with an average abundance > 1% in the 16S rRNA gene amplicon sequencing (entire) are shown.

**
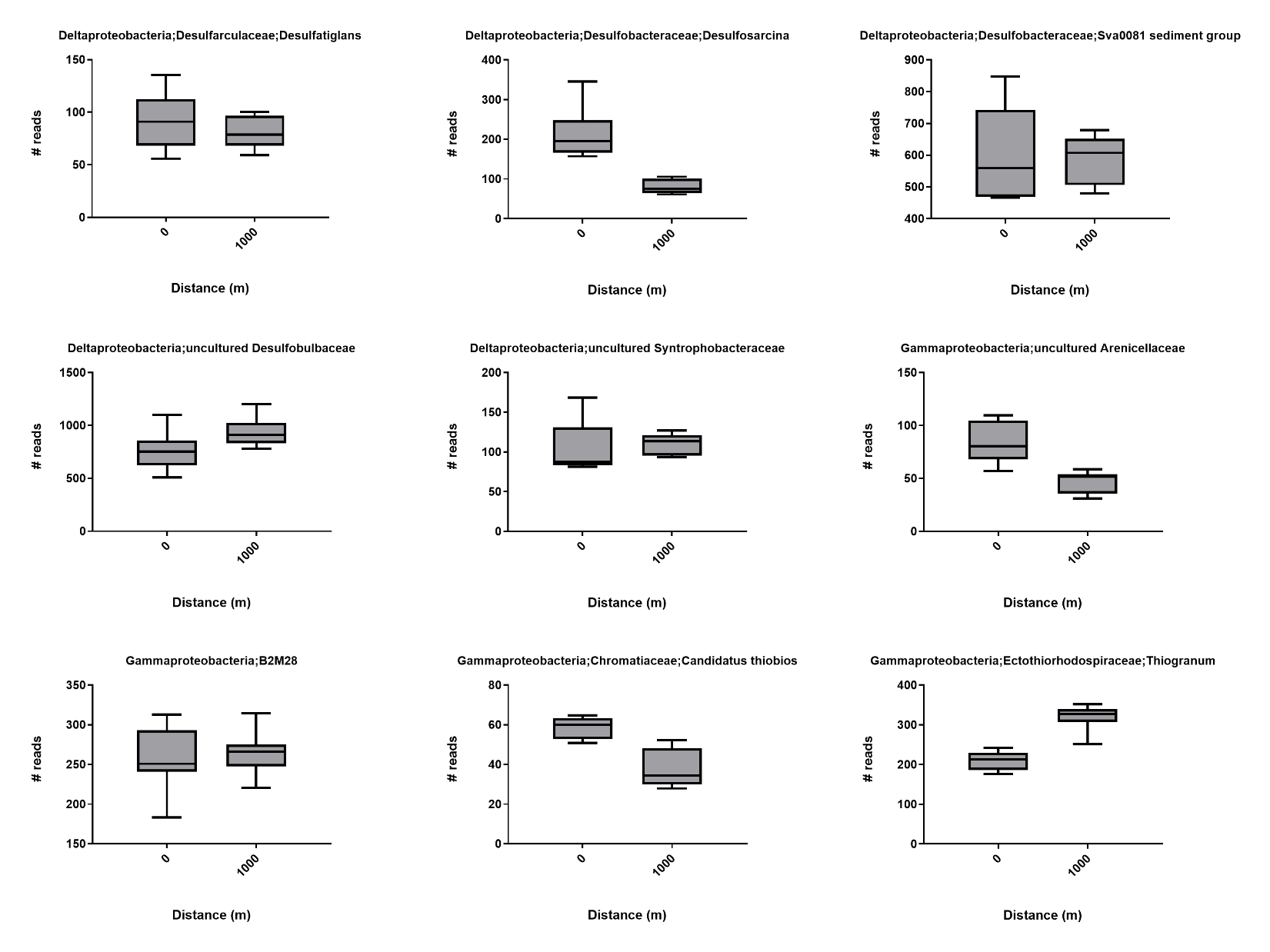
**

**Figure S4 cont.: Boxplots showing mean (min/max) of** **abundant bacterial genera in embayments.** Only genera with an average abundance > 1% in the 16S rRNA gene amplicon sequencing (entire) are shown.

**
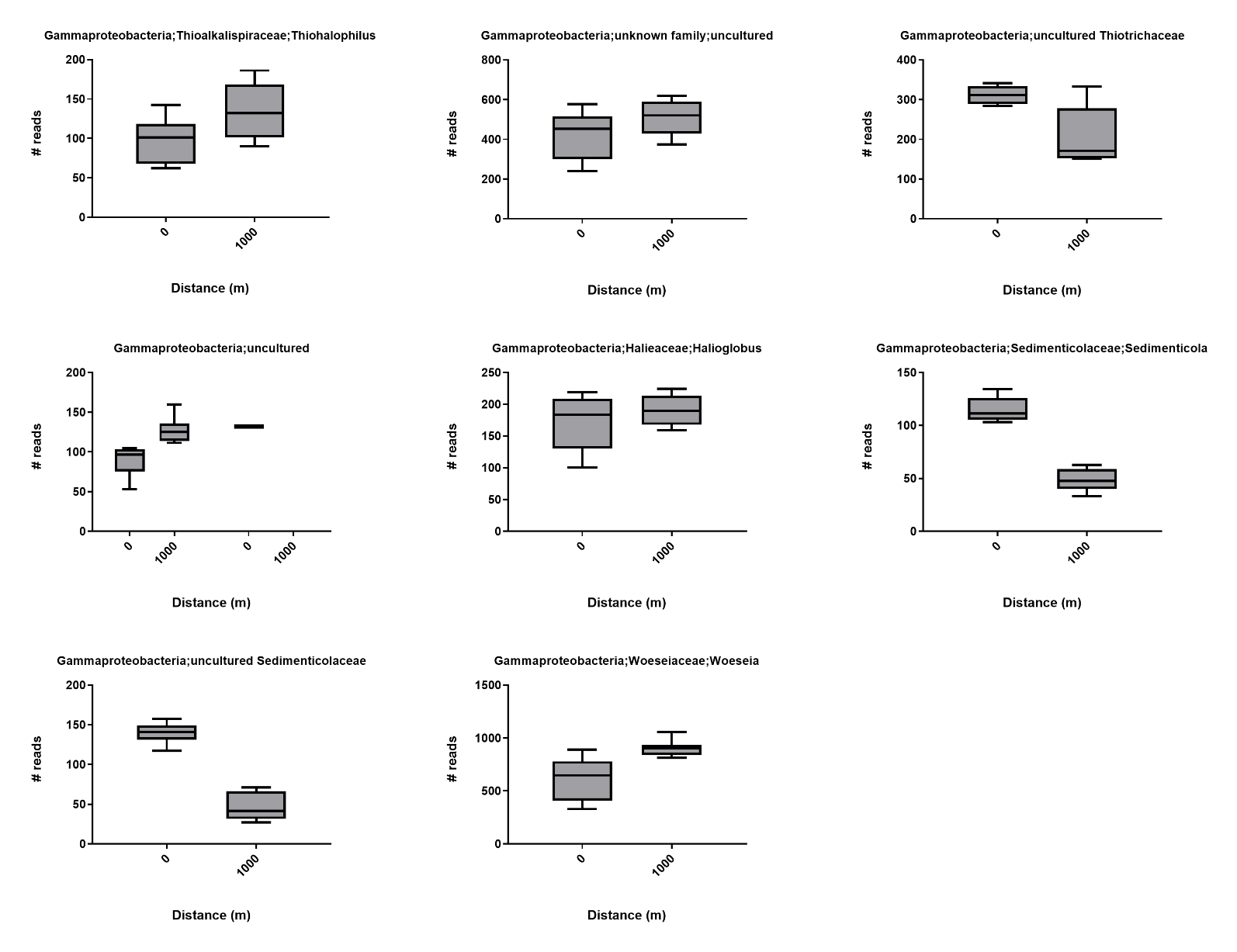
**

**Figure S4 cont.: Boxplots showing mean (min/max) of** **abundant bacterial genera in embayments.** Only genera with an average abundance > 1% in the 16S rRNA gene amplicon sequencing (entire) are shown.


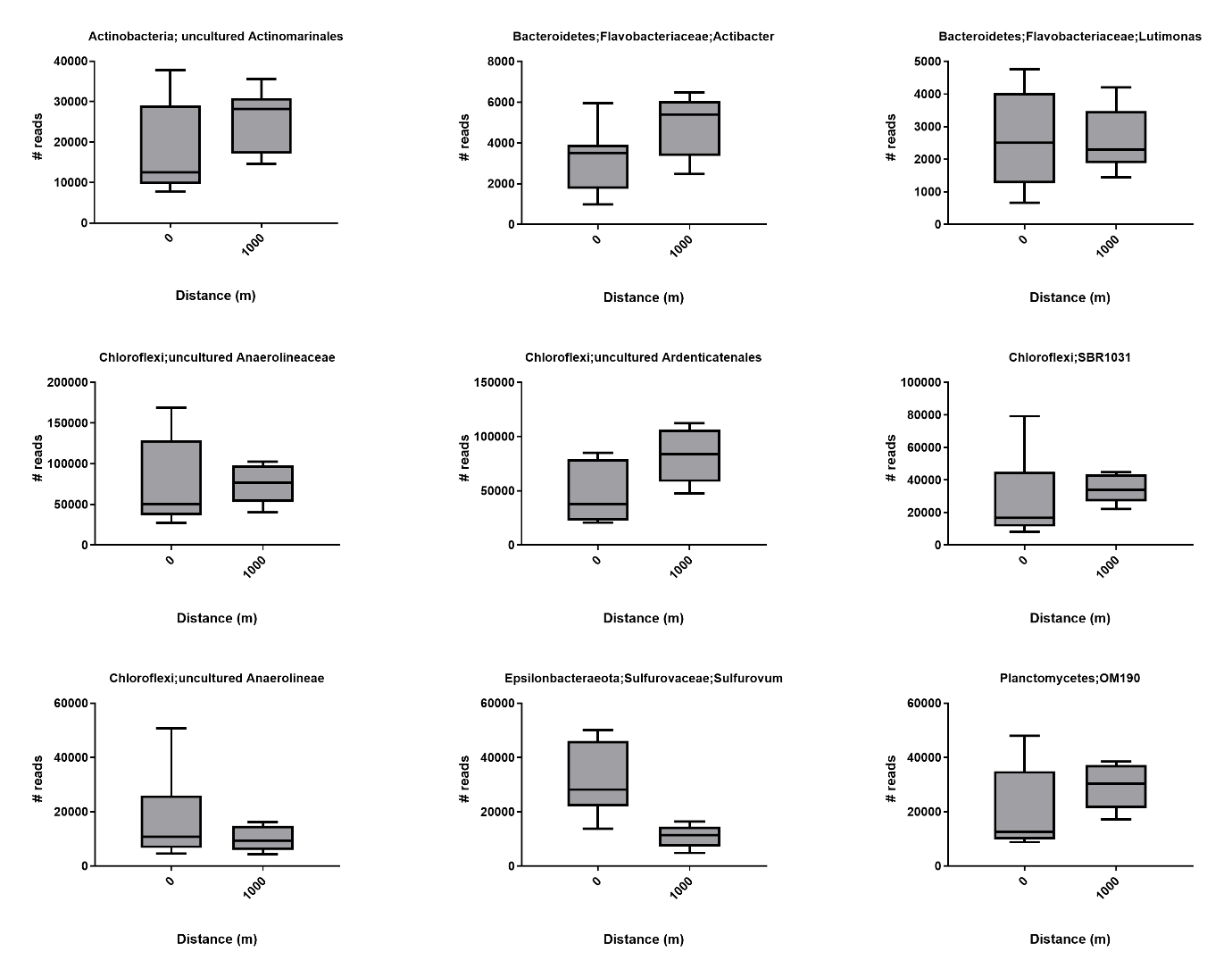


**Figure S5: Boxplots showing mean (min/max) of** **abundant bacterial genera in embayments.** Only genera with an average abundance > 1% in the rRNA (active) dataset, respectively are shown.


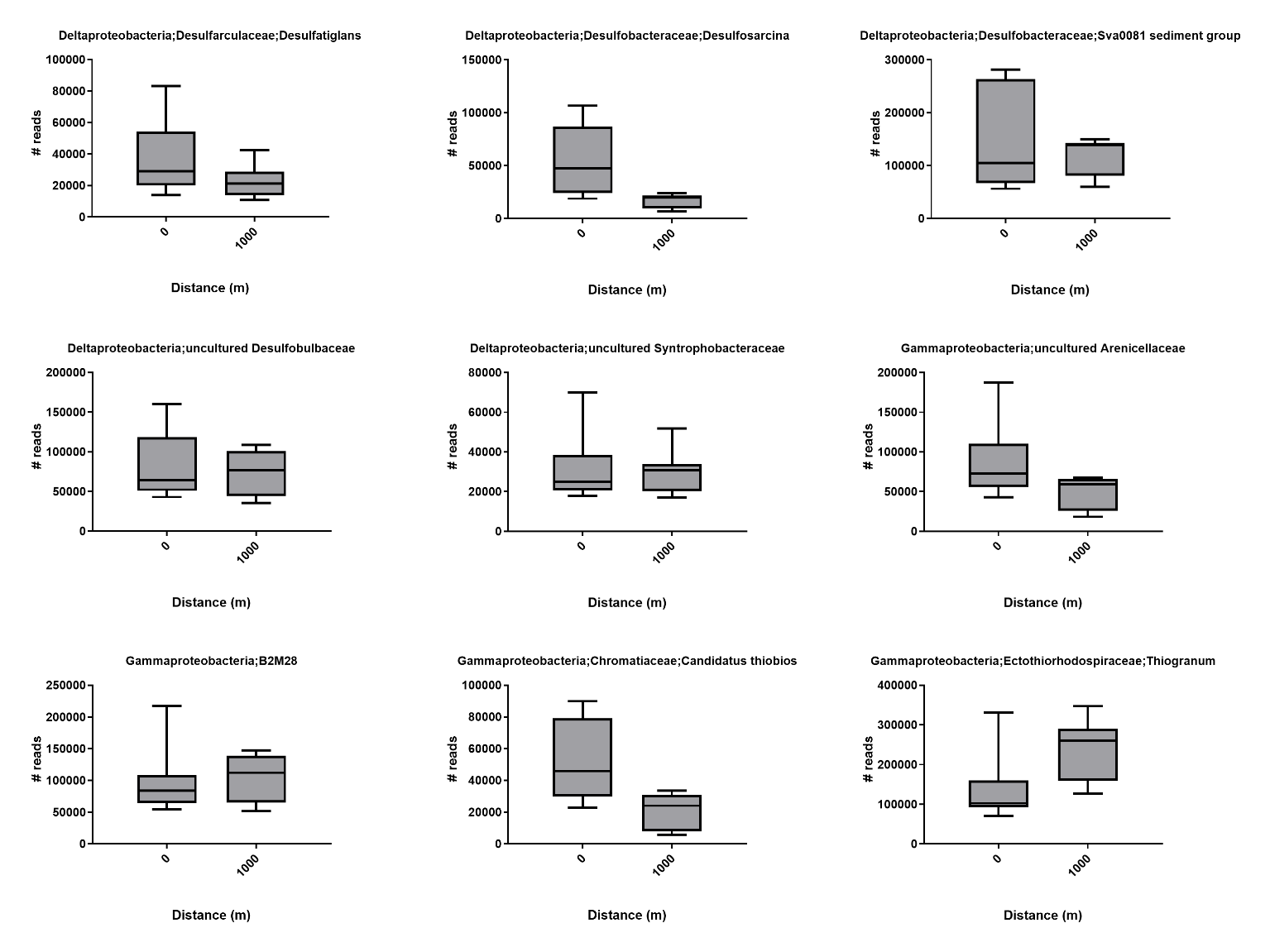


**Figure S5 cont.: Boxplots showing mean (min/max) of** **abundant bacterial genera in embayments.** Only genera with an average abundance > 1% in the rRNA (active) dataset, respectively are shown.

**
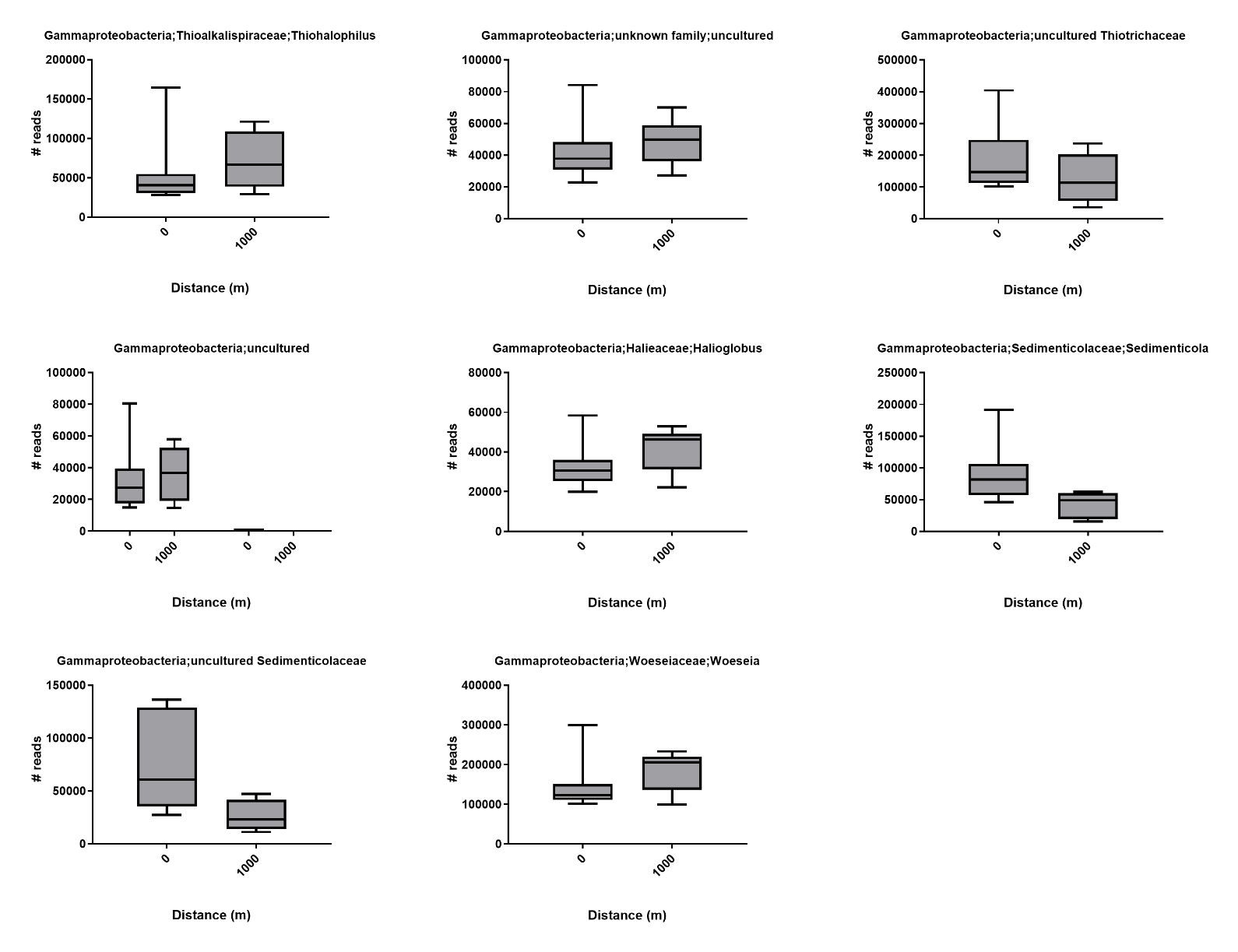
**

**Figure S5 cont.: Boxplots showing mean (min/max) of** **abundant bacterial genera in embayments.** Only genera with an average abundance > 1% in the rRNA (active) dataset, respectively are shown.


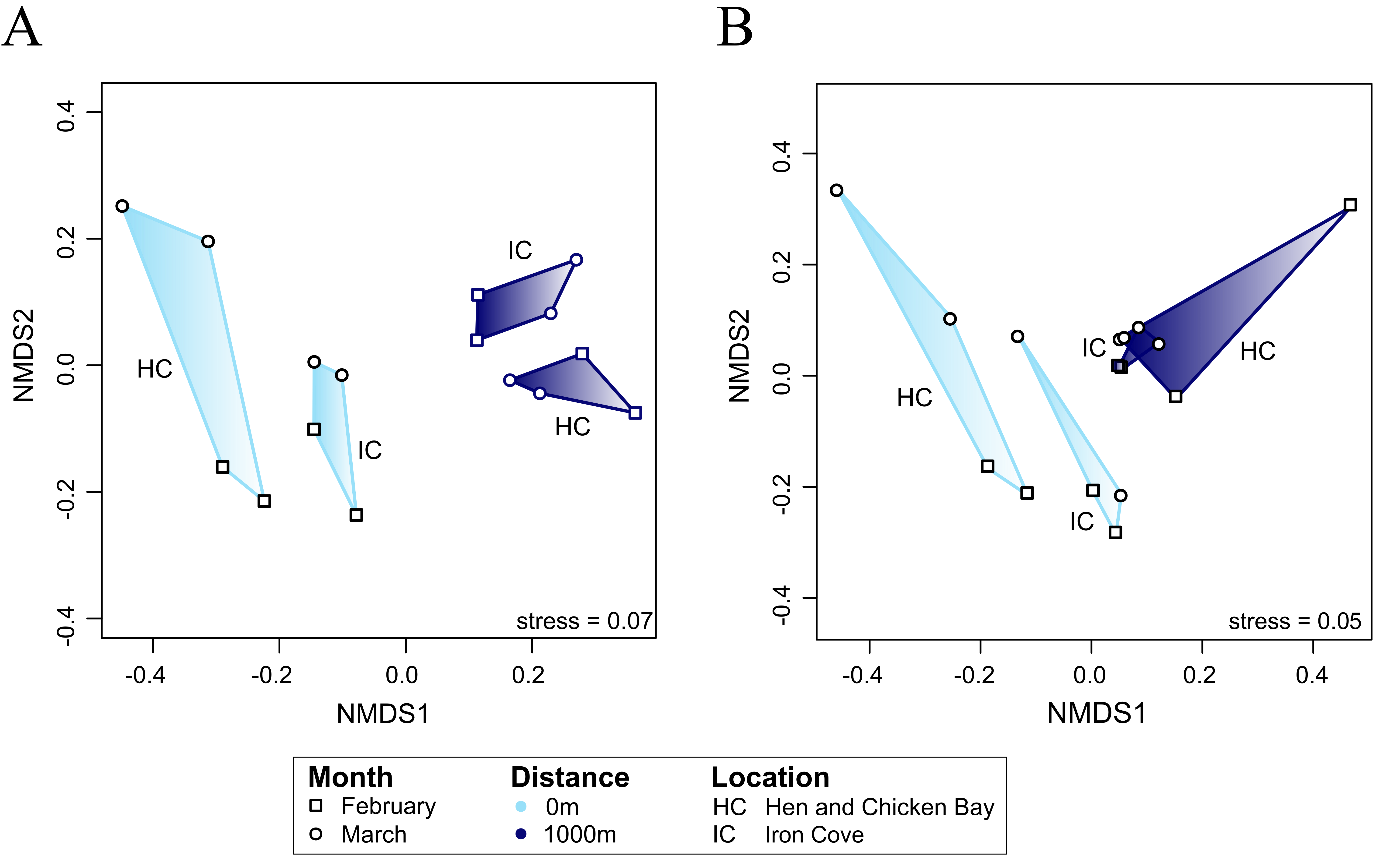


**Figure S6**: **Ordination analysis based on the composition of all assembled non-rRNA transcripts (A) and of those potentially encoding bacterial proteins (B).** The assembly of the non-rRNA was performed with rnaSPAdes. Ordination is based on Bray-Curtis dissimilarities between samples and is color-coded by distance. Different symbols represent the two sampling months. Data included in Supplementary Table S4.
